# A large population of diverse neurons in the *Drosophila *central nervous system expresses short neuropeptide F, suggesting multiple distributed peptide functions

**DOI:** 10.1186/1471-2202-9-90

**Published:** 2008-09-19

**Authors:** Dick R Nässel, Lina E Enell, Jonathan G Santos, Christian Wegener, Helena AD Johard

**Affiliations:** 1 Department of Zoology, Stockholm University, S-10691 Stockholm, Sweden; 2 Emmy Noether Neuropeptide Group, Department of Biology, Animal Physiology, Philipps-University, D-35032 Marburg, Germany

## Abstract

**Background:**

Insect neuropeptides are distributed in stereotypic sets of neurons that commonly constitute a small fraction of the total number of neurons. However, some neuropeptide genes are expressed in larger numbers of neurons of diverse types suggesting that they are involved in a greater diversity of functions. One of these widely expressed genes, *snpf*, encodes the precursor of short neuropeptide F (sNPF). To unravel possible functional diversity we have mapped the distribution of transcript of the *snpf *gene and its peptide products in the central nervous system (CNS) of *Drosophila *in relation to other neuronal markers.

**Results:**

There are several hundreds of neurons in the larval CNS and several thousands in the adult *Drosophila *brain expressing *snpf *transcript and sNPF peptide. Most of these neurons are intrinsic interneurons of the mushroom bodies. Additionally, sNPF is expressed in numerous small interneurons of the CNS, olfactory receptor neurons (ORNs) of the antennae, and in a small set of possibly neurosecretory cells innervating the corpora cardiaca and aorta. A sNPF-Gal4 line confirms most of the expression pattern. None of the sNPF immunoreactive neurons co-express a marker for the transcription factor DIMMED, suggesting that the majority are not neurosecretory cells or large interneurons involved in episodic bulk transmission. Instead a portion of the sNPF producing neurons co-express markers for classical neurotransmitters such as acetylcholine, GABA and glutamate, suggesting that sNPF is a co-transmitter or local neuromodulator in ORNs and many interneurons. Interestingly, sNPF is coexpressed both with presumed excitatory and inhibitory neurotransmitters. A few sNPF expressing neurons in the brain colocalize the peptide corazonin and a pair of dorsal neurons in the first abdominal neuromere coexpresses sNPF and insulin-like peptide 7 (ILP7).

**Conclusion:**

It is likely that sNPF has multiple functions as neurohormone as well as local neuromodulator/co-transmitter in various CNS circuits, including olfactory circuits both at the level of the first synapse and at the mushroom body output level. Some of the sNPF immunoreactive axons terminate in close proximity to neurosecretory cells producing ILPs and adipokinetic hormone, indicating that sNPF also might regulate hormone production or release.

## Background

Insect neuropeptides are commonly expressed in discrete stereotypic populations of neurons in the central nervous system [1-7]. These populations vary in size for different neuropeptides. Thus, some *Drosophila *neuropeptides such as SIFamide and eclosion hormone are found in very small numbers of uniform neurons [8-11], whereas others like tachykinin-related peptides (TKRPs), proctolin, and myoinhibitory peptides (MIPs) are expressed in large populations of diverse neurons [7,12,13]. A closer look at the expression of different insect neuropeptides has revealed that some peptides are prominent in neurosecretory cells and other endocrine cells and play roles as circulating hormones (see [3,14,15]). Many peptides are found both in endocrine systems and in interneurons and thus they are likely to have several functions, including modulatory actions in central circuits (reviewed in [3,16]). A few peptides can additionally be seen in skeletal or visceral motoneurons [17,18]. Finally, some *Drosophila *neuropeptides appear to be exclusively expressed in interneurons, for example the TKRPs, SIFamide and A-type allatostatins [8,12,19], but there are so far no clear demonstrations of identified insect neuropeptides in sensory neurons (see [3,16]). Taken together the available data suggest that neuropeptides can act in diverse ways as neuromodulators or cotransmitters in the CNS, as circulating hormones and as neuromediators released by neurons at peripheral targets. Thus, one may ask to what extent a neuropeptide that is widely distributed in many types of neurons is multifunctional. Or, differently phrased, does a given peptide expressed in a diverse set of neuronal circuits and neuron types (including neurosecretory cells) subserve a global unified function or a multitude of separate distributed functions? To address this question we analyzed a *Drosophila *peptide gene with very wide distribution.

Recently it was demonstrated that the *Drosophila *gene, *snpf*, that encodes the predicted peptides short neuropeptide F 1 – 4 (sNPF 1 – 4), is expressed by numerous neurons of the *Drosophila *brain [20,21]. The study by Johard et al. [21] focused on the presence of *snpf *in Kenyon cells of the mushroom bodies, known to be the intrinsic components of this brain center subserving, among other things, higher olfactory processing and learning [22,23]. During the analysis of the mushroom body circuits it was noted that *snpf *transcript and peptide products are distributed in a large population of different types of neurons throughout the *Drosophila *CNS. Thus we decided to map the distribution of sNPFs in relation to other neurotransmitters, neuronal circuits and compartmental boundaries (segments/neuromeres) of the CNS to allow analysis of substrates for possible diversity of functional roles.

We made a detailed mapping of sNPFs, especially in interneurons of various brain centers in larvae and adult *Drosophila*. For immunocytochemical charting we used specific antisera to two of the peptide products of the *snpf *gene as well as a sequence of the precursor protein. This was compared with *in situ *hybridization with a ribonucleotide probe to the *snpf *transcript. A sNPF-Gal4 line was used for driving green fluorescent protein (GFP) and the labeling pattern compared to sNPF immunolabeling. To screen for colocalized neurotransmitters and to analyze relations to identified structures we mapped sNPF expression in relation to specific neurons identified by Gal4 driven GFP. This analysis included the relation between sNPF expression and that of the transcription factor DIMMED (DIMM), known to be present in secretory peptidergic neurons [15,24]. A fasciclin-2 (Fas2) marker was utilized to relate neurons to axon tracts and segmental compartments [7,25]. One of the major findings is that *snpf *products are expressed in a very large number of neurons of diverse types (some of which co-express markers for various small molecule neurotransmitters) suggesting multiple functions of these peptides. No overlap was seen between sNPF and DIMM expression. A novel and exciting finding was the presence of sNPF in a subpopulation of the olfactory receptor neurons of the antennae and in a pair of abdominal projection neurons co-expressing insulin-like peptide 7 (ILP7).

## Results

### Distribution of sNPF in the larval CNS

#### In situ hybridization in larval CNS

A general description of the distribution of *snpf *transcript in the embryonic and larval CNS has been provided earlier [20]. Here we provide a more detailed localization of transcript by *in situ *hybridization in the brain and ventral cord of the third instar larva. Six larval CNS were used for analysis of hybridization pattern.

**Figure 1:**
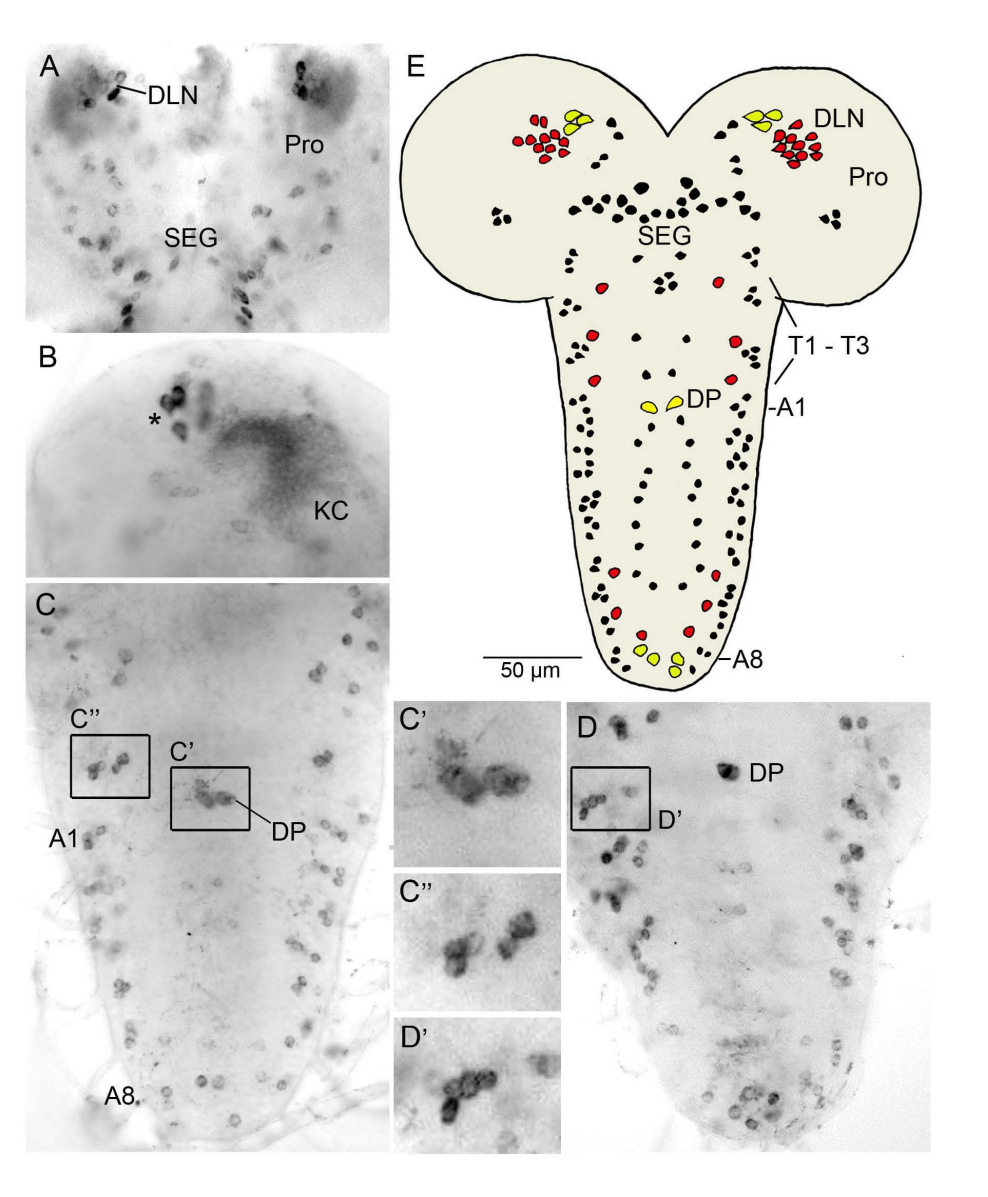
**Cell bodies of *snpf*/sNPF expressing neurons in the larval CNS.***In situ *hybridization reveals *snpf *transcript in A-D and immunocytochemistry displays cell bodies in E. **A**. *In situ *signal in neurons of the protocerebrum (Pro) of the brain and subesophageal ganglion (SEG). DLN, dorsolateral neurons (including possible neurosecretory cells). **B**. Higher magnification of the right brain hemisphere in another specimen reveals numerous Kenyon cells (KC) of the mushroom body and three of the strongly labeled cell bodies dorsally (asterisk) that may represent neurosecretory cells. **C and D**. In the ventral nerve cord segmentally arranged cell bodies display *in situ *signal. Eight abdominal neuromeres are indicated (A1 – A8). The boxed areas are shown in higher magnification in C', C" and D': in C" and D' lateral cell body clusters are shown with 4 – 5 neurons in each hemineuromere and in C' the two dorsal cell bodies (DP) of A1 are visible. **E**. Tracing of cell bodies labeled with antiserum to sNPF precursor-derived peptide (sNPFp). In this tracing only strongly labeled cell bodies are depicted and the Kenyon cells were ommitted. Additional small cell bodies with variable labeling intensity can be seen in brain and ventral nerve cord. Red cell bodies are dorsal, black ones ventral or lateral and yellow ones dorsal and with larger diameters (yellow cellbodies in the brain probably correspond to the ones indicated with asterisk in B). Two groups of cell bodies are indicated, DLN and DP. T1 – T3, thoracic neuromeres, other abbreviations as in A – D.

In the larval brain and subesophageal ganglion there are about 35 neurons in each hemisphere expressing *in situ *signals, in addition to numerous Kenyon cells (see [21]). These neurons are located dorsally and ventrally in the protocerebrum and in the subesophageal ganglion (Fig. 1A, B). Three strongly in situ labeled neurons in each protocerebrum have larger cell bodies (Fig. 1A, B, see also 1E) and are likely to be neurosecretory cells (see next section). In the ventral nerve cord there are segmentally distributed neurons both in thoracic and abdominal neuromeres (Fig. 1C, D). In each side of the thoracic and abdominal neuromeres there are three to four lateral cell bodies and one dorso-median cell body (Fig. 1C, D). In abdominal neuromeres A7 and A8 there are additional labeled cell bodies with larger diameters (Fig. 1D). A characteristic pair of larger cell bodies (DP) can be seen dorsally and medially in A1 (Fig. 1C, D). The segmental distribution and numbers of in situ labeled neurons match the pattern displayed by sNPF immunolabeling (see Fig. 1E).

#### Immunocytochemistry in larval CNS

The immunolabeling with antiserum to the sNPF precursor derived peptide (sNPFp) was found most consistent and reliable. This antiserum recognizes a peptide sequence not shared with other known neuropeptides and thus presumably only labels neurons producing the sNPF precursor. Also the antiserum to the RLRWamide sequence is likely to only bind to sequences present on the sNPF precursor (the predicted sNPF-3 and 4) [21]. On the other hand the antiserum to sNPF-1_4–11 _(with a RLRFamide C-terminus) is likely to cross react also with neurons expressing peptides with RFamide in the C-terminus such as: long NPF (dNPF), extended FMRFamides (dFMRFa), myosuppressin (DMS) and sulfakinins (DSKs). Thus after confirming that the antisera to sNPFp and RLRWamide labeled what appears to be the same neuron populations and that the antiserum to RLRFamide labels these neurons plus additional ones [21], we decided to use the sNPFp antiserum throughout the present investigation. This antiserum was characterized for specificity by Johard et al. [21]. It can be mentioned that in the investigation by Lee et al. [20] an antiserum to a full-length sNPF-2 was used. That antiserum is likely to cross react with other RFamide-containing neuropeptides, and indeed these authors describe neurons additional to those shown here.

The number of neuronal cell bodies given below refers to both hemispheres (unless specified). One characteristic of the sNPFp immunolabeling is a variability in labeling intensity, especially in smaller neurons. Furthermore the labeling of neuronal processes is punctate and thus it is hard to resolve the axonal projections and therefore individual neurons could not be fully reconstructed morphologically. We examined sNPFp immunolabeling patterns in at least 10 specimens of each stage. It can be noted that the sNPFp immunlabeling pattern of cell bodies, closely resembles that seen after snpf in situ hybridization.

**Figure 2:**
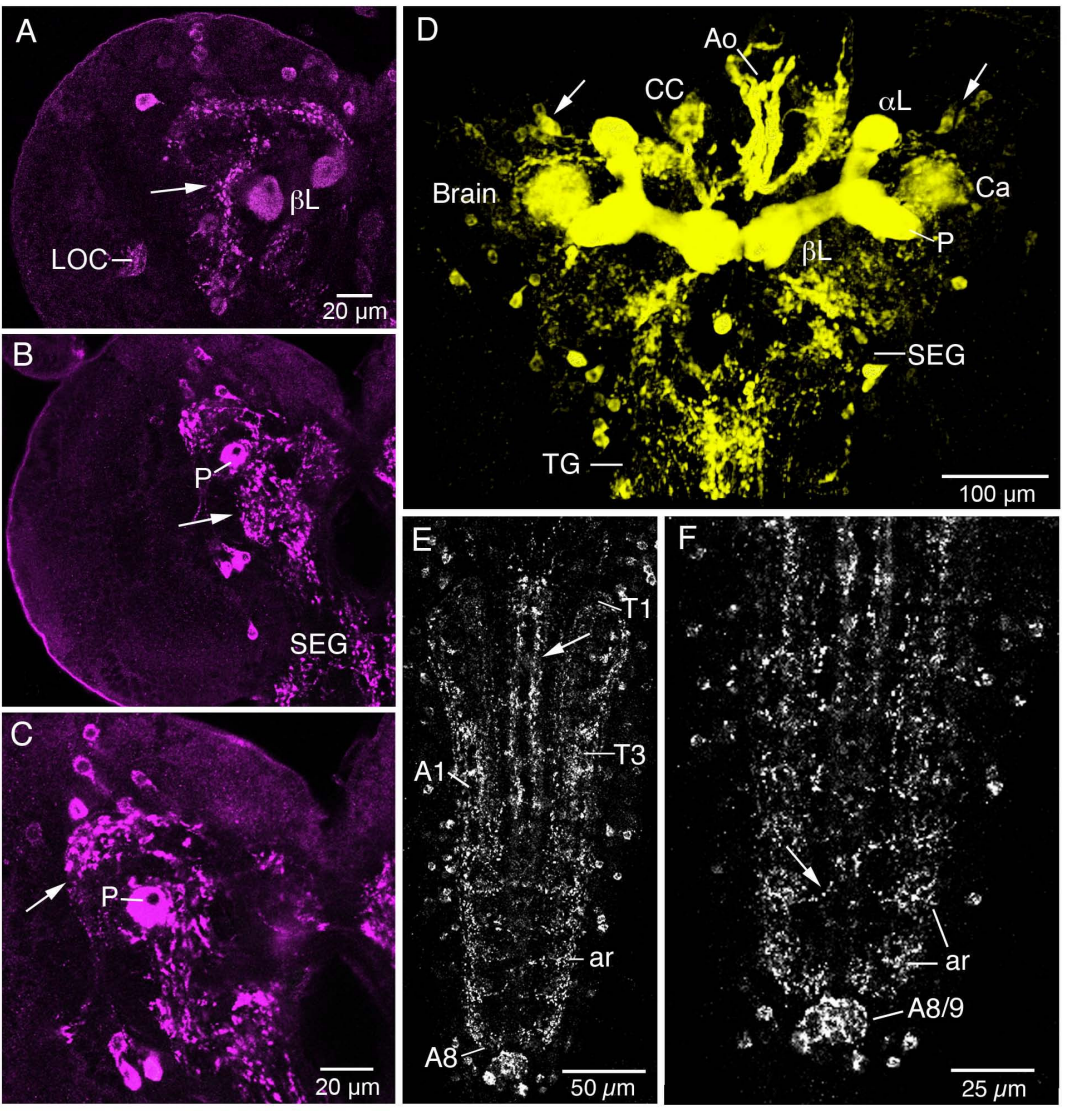
**Immunolabeling with antiserum to sNPFp in the larval CNS. Horizontal views, anterior is to the top in all images. **A – C**.** Three different levels of the left brain hemisphere displaying immunolabeled neuronal cell bodies and processes in neuropils (e. g. at arrows). LOC, larval optic center; βL, β-lobe and P, peduncle of mushroom body; SEG, subesophageal ganglion. **D**. A threshold-based 3D reconstruction of the brain, subesophageal ganglion (SEG) and first part of thoracic ganglia (TG). Note strong labeling of mushroom body lobes and peduncle (αL, βL, P) and axonal projections to the anterior aorta (Ao) and corpora cardiaca (CC) of the ring gland. The mushroom body calyx (Ca) is less intensely labeled. A bilateral cluster of cell bodies (at arrows) contain the lateral neurosecretory cells supplying axons to the ring gland. **E and F**. Confocal sections of immunolababeling in the ventral nerve cord with thoracic (T1 – T3) and abdominal (A1 – A8/9) neuromeres. Punctate sNPFp immunosignals are seen in neuropils laterally (ar) and medially, as well as in longitudinal tracts (e. g. at arrow in E) and segmental commissures tracts (e. g. at arrow in F). Immunolabeling is also seen in the terminal plexus in A8 and A9.

**Figure 3:**
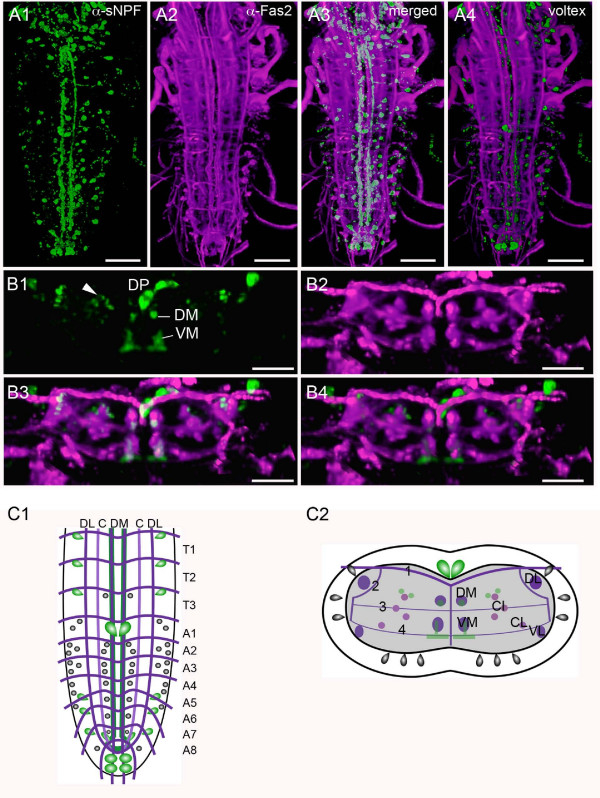
**Relation between sNPFp immunolabeled structures and fasciclin2 (Fas2)-immunolabeled tracts/boundaries in the larval ventral cord.** These are voltex reconstructions from confocal image stacks. **A1 – A4**. Specimen double labeled with sNPFp (green) and anti-Fas2 (magenta) reveals segmental organization of cell bodies and relation of sNPFp-IR axon tracts to Fas2 tracts. In A1–A3 maximum projections are shown and in A4 a voltex reconstruction (see methods). **B1 – B4**. A cross section through first abdominal segment (neuromere A1) showing relations between sNPF labeled structures and Fas2 tracts. The dorsal median cell bodies of A1 are visible (DP) as well as axons in the major ventral median tracts (VM and DM; see C2) and along the C1 tract (arrow head). **C1**. Schematic presentation of sNPFp labeled neurons (grey and green) and Fas2 tracts (magenta) revealing segmental localization of cell bodies, dorsal view. **C2**. Transverse view of A1 showing sNPFp-IR cell bodies (grey and green) and axons (green) in relation to Fas2 labeled tracts (magenta). Grey: small cells with variable and weaker staining intensity, Green: larger cells with consistent and strong staining intensity. The longitudinal tracts are designated DL (dorso-lateral), DM (dorso-medial), VM (ventro-medial), CI (centro-intermediate), CL (centro-lateral) and VL (ventro-lateral) and transverse tracts 1 – 4. Note that cell bodies located dorsally, laterally and ventrally. Scale bars: 50 μm in A1–A4, 30 μm in B1–B4.

There are up to 600 Kenyon cells per larval hemisphere [26]. As shown earlier [21] most of these appear to be sNPFp-immunoreactive (sNPFp-IR). In addition there are about 190 – 200 sNPFp-IR neuronal cell bodies in the larval CNS (as summarized in Fig. 1E). Of these about 70 are in the brain, including the subesophageal ganglion, and a minimum of 120 consistently labeled neurons in the thoracic and abdominal neuromeres (Fig. 1E, 2, 3). There is some variability in the number of small neurons labeled with the sNPFp antiserum, both in the brain and the ventral ganglia. Thus, in well-labeled specimens we can observe additional small cell bodies, especially in the abdominal ganglia.

The sNPFp-IR neuronal cell bodies in the larval brain and subesophageal ganglion are distributed in several bilateral clusters: around 20 – 26 cell bodies are located in the subesophageal ganglion and the remaining ones in different parts of the protocerebrum (Fig. 1E, 2A–D). Three pairs of intensely immunolabeled cells dorsally in the protocerebrum are likely to be neurosecretory cells (LNCs) of the pars lateralis (Fig. 1E, 2D). These LNCs have axons terminating in the ring gland (corpora cardiaca portion) and in the anterior aorta. We will describe these neurosecretory cells and their neurohemal release sites in more detail below.

In the larval brain branching sNPFp-IR processes were seen in neuropils of the protocerebrum, deutocerebrum and subesophageal ganglion (Fig. 2A–D). The most dense immunolabeling is in the lobes of the mushroom bodies (Fig. 2A, C, D). Further sNPFp immunolabeling of processes is seen in the larval optic center (Fig. 2A) and in non-glomerular neuropils surrounding mushroom body lobes and in pars intercerebralis and lateralis (Fig. 2A–D). A movie with a rotating view of the brain is shown in Additional File 1.

The general distribution of sNPFp-IR neuronal cell bodies in thoracic and abdominal neuromeres is shown in Fig. 1E and 3. These cell bodies are of three general size classes: small, intermediate and larger. There are only six larger and 12 intermediate sized cell bodies, the remaining ones are small. As mentioned, the variable labeling is only seen for the small cell bodies.

The cell bodies in thoracic and abdominal neuromeres are located dorsally, ventrally and laterally; the former two either along the midline or more laterally (Fig. 1E, 3). By double labeling with antisera to sNPFp and fasciclin 2 (fas2), that labels major axonal tracts [7,25], we could relate the cell bodies to the different neuromeres of the ventral ganglia (Fig. 3). From this we could reveal that in each of the three thoracic neuromeres there is only one pair of neurons with intermediate sized cell bodies. However, there are up to four additional pairs of neurons with small lateral cell bodies in each of the thoracic neuromeres. Also medially there are at least two small ventral neurons per neuromere.

The abdominal neuromeres display sNPFp immunoreactivity in many small neurons laterally and ventrally (Fig. 1E, 2E, F). In A1 – 4 there are at least 4 – 6 lateral small neurons and 2 – 4 median/ventral ones per neuromere (Fig. 1E, 3). A5 – A8 have 2 – 6 lateral and 2 median small cell bodies (Fig. 1E, 3). In some specimens these posterior neuromeres appear to lack lateral small neurons (or display weak label). Finally, in each of A6 – 8 there is a pair of intermediate size cell bodies laterally. Two groups of large sized abdominal neurons stand out as individually identifiable: one pair dorsally in A1 (DP) and two pairs posteriorly in A8 (see Fig. 1E, 2E, F, 3B, C).

Two pairs of major bundles of sNPR-IR axons were found in the ventral ganglia. These are located ventrally and medially in the ganglia, and are associated with the dorso-median (DM) and ventro-median (VM) tracts (Fig. 2E, 3). The axons in the VM tracts form numerous varicosities and dorso-ventral branches along their course (see movie in Additional File 2). Additionally two pairs of thin lateral tracts near the CI tracts contain sNPFp-IR axons (Fig. 3C2). sNPFp-IR punctates are seen in several neuropil areas both dorsally and ventrally in the thoracic and abdominal neuromeres (Fig. 2E, F). The delineated neuropil posteriorly in A8/9 (designated terminal plexus [7]) is densely supplied with sNPFp-IR punctates (Fig. 2F). We detected no clear cases of sNPFp-IR axons in commissural tracts of thoracic and abdominal neuromeres, but immunoreactive punctae could be seen in transverse bands in each neuromere (Fig. 2E, F). None of the neurons in the thoracic and abdominal neuromeres seem to send axons through peripheral nerve roots, suggesting that they are all interneurons. A view of sNPF-IR neurons in the ventral nerve cord is shown in the movie in Additional File 2.

### Expression of sNPF-Gal4 in the larval CNS

**Figure 4:**
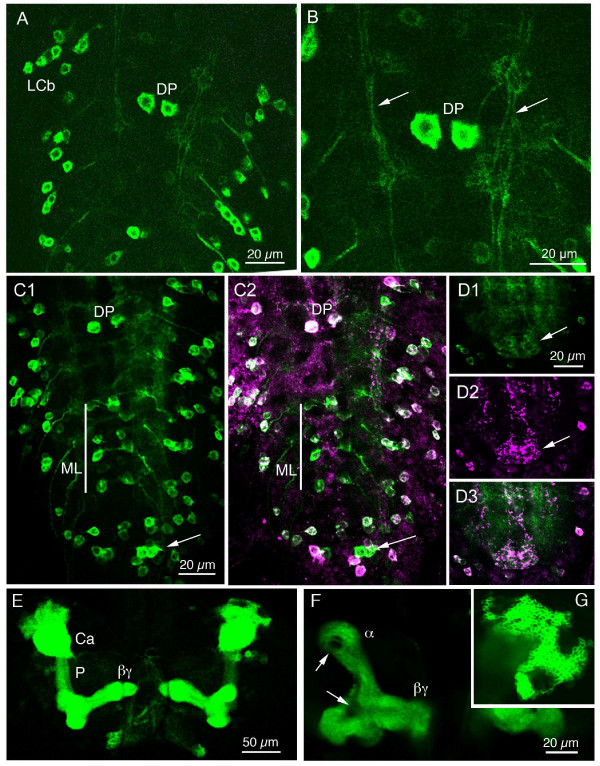
**Expression of sNPF-Gal4 driven GFP in the larval CNS. The NP6301 Gal4 line appears to match the sNPF immunolabeling as seen in these dorsal views of the ventral nerve cord (A-D and brain (E-G).** Anterior is up in the images. **A**. sNPF-Gal4 expression in third thoracic and first three abdominal neuromeres. Segmentally arranged lateral cell bodies (LCb) and the dorsal pair (DP) in A1. **B**. Detail of the DP neurons and longitudinal axonal tracts (arrows). **C1 and C2**. Double labeling with sNPF-Gal4-GFP and anti-sNPFp in abdominal neuromeres. Note that with a few exceptions the two markers colocalize. A set of median segmental neurons (ML) and some posterior neurons (arrow) express only sNPF-gal4. **D1-3**. Expression of immunolabeling and Gal4 in processes of the terminal plexus in A8/9 (arrows). E-G. In the larval brain the intrinsic neurons of the mushroom bodies express sNPF-Gal4. GFP can thus be seen in α,β and γ-lobes, the peduncle (P) and calyx (Ca). Note that the core of the lobes is devoid of GFP (arrows). In G the cell bodies of the Kenyon cells are depicted.

To our knowledge this is the first report on the expression pattern of the sNPF-Gal4 (NP6301). Using this sNPF-Gal4 in third instar larvae we found a GFP expression pattern similar to the distribution of sNPFp-IR neurons (Fig. 4). Double labeling with sNPFp antiserum indeed indicated that most if not all neurons in the thoracic and abdominal ganglia expressed both markers (Fig. 4C, D). A set of segmental dorsal median cell bodies in abdominal neuromeres display sNPF-Gal4, but not sNPF-IR material (Fig. 4C). In the brain the match was not complete, probably due to low expression levels of GFP in some neurons (not shown). There were also some neurons where GFP was seen, but not immunolabeling; probably caused by the variable immunolabeling seen in some of the smaller neurons.

In the ventral nerve cord we could detect the typical pattern of sNPF expressing cell bodies, but now also the details of their axonal projections and arborizations (Fig. 4A – C). It is obvious that sNPF expressing neurons supply processes to several neuropil areas in the thoracic and abdominal ganglia, as already indicated with less clarity by immunolabeling. We detected no efferent or afferent axons in the ventral nerve cord suggesting that all sNPF expressing neurons in this region are interneurons.

In the larval brain the most prominent sNPF-Gal4 expression was seen in Kenyon cells of the mushroom bodies (Fig. 4E). The entire neuronal morphology of these Kenyon cells was revealed and it was clear that some neurons in the core of the calyx and lobes do not express GFP (Fig. 4F, G), similar to the sNPFp antiserum labeling [21]. This Gal4 line also enabled us to see the neurosecretory cells supplying axons to the ring gland (corpora cardiaca), as will be described below. Sets of Gal4 expressing axons were seen in the nerves entering the larval olfactory centers in the brain. These may be subsets of axons from ORNs of the dorsal organs as also suggested from in situ labeling in the late embryo [20].

### Supply of sNPF expressing axons to the ring gland and aorta

**Figure 5:**
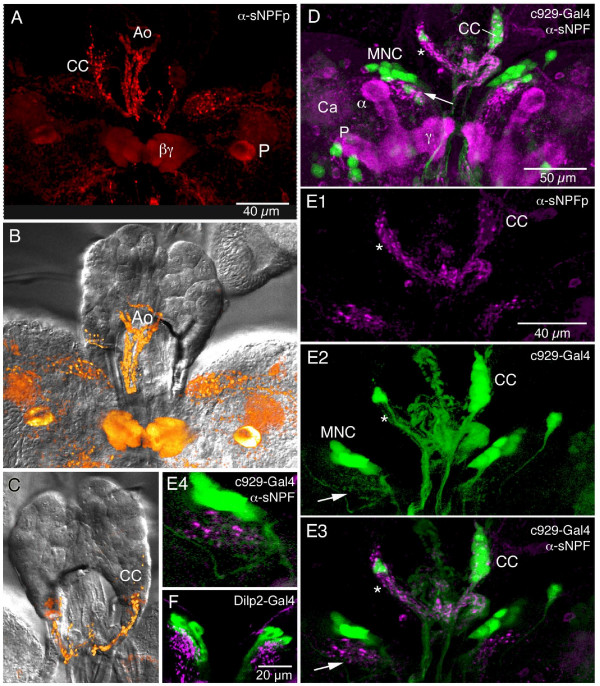
**Distribution of sNPF precursor immunoreactivity (sNPFp-IR) in relation to the ring gland and anterior aorta of the larva (anterior is up).A**. sNPFp-IR (red) in brain and the corpora cardiaca (CC) portion of the ring gland and aorta (Ao). Immunolabeling is also seen in peduncle (P) and β and γ-lobes of the mushroom bodies. **B and C**. Dorsal (**B**) and ventral (**C**) aspects of retrocerebral complex in superimposed Nomarsky optics and immunofluorescence. In **B **the sNPFp-IR processes on the aorta are seen and in **C **the axons in the CC part of the ring gland. **D**. Double labeling with c929-Gal4-driven GFP (to display DIMM expression) and anti-sNPFp (magenta) shown from stack of confocal images. The c929 displays AKH-producing cells in the CC and median neurosecretory cells (MNC) in the pars intercerebralis some of which produce insulin-like peptides. Note sNPFp-IR axons invading the CC (asterisk) and a region adjacent ot the MNCs (arrow). The mushroom body calyx (Ca), peduncle (P) and lobes (α and γ) are indicated. **E1 – E4**. Confocal sections with details of sNPFp-IR fibers invading the corpora cardiaca portion of the ring gland. In **E1 **sNPFp processes are shown and in **E2 **c929-Gal4-driven GFP in neurosecretory cells in the CC and pars intercerebralis (MNC). Note dendritic branches of the neurosecretory cells (arrow). **E3 **shows the merged image with superposition of sNPFp-IR processes with endocrine cells in corpora cardiaca (CC and asterisk) and branches at arrow. **E4 **An enlarged detail of the dendrites of the MNCs in E3 (left hemisphere) with impinging sNPF-IR terminations. **F **Using a Dilp2-Gal4 to drive GFP it can be seen that the sNPF terminations superimpose on processes of the Dilp2-expressing neurons.

**Figure 6:**
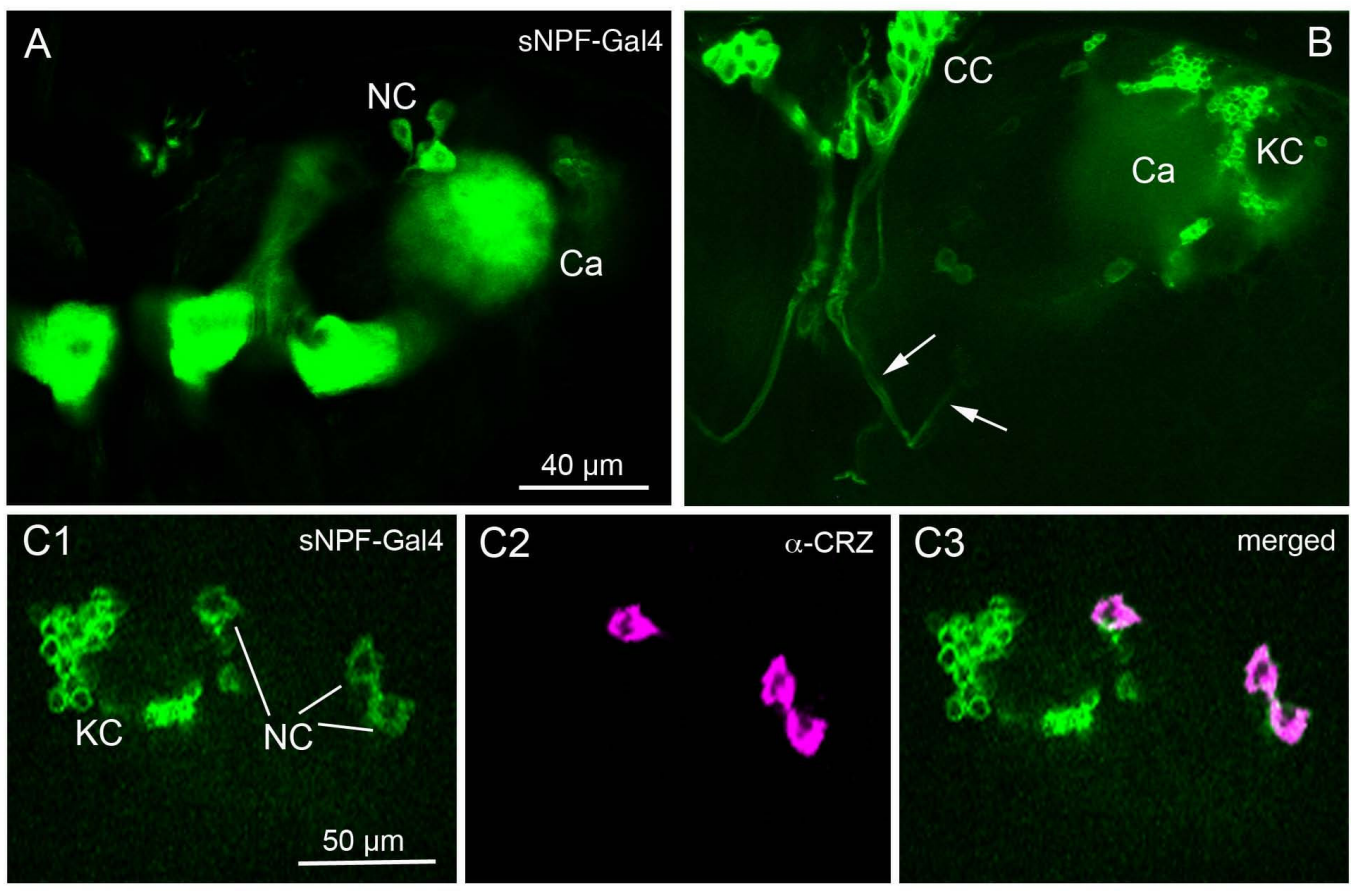
**Neurosecretory cells that express sNPF and corazonin.A**. The source of the sNPF-Gal4 expressing axon terminations in the ring gland can be traced to a set of three neurons (NC) dorsal to the mushroom body calyces (Ca). **B**. The axons from the three neurosecretory cells (arrows) can be followed to the corpora cardiaca (CC). In the CC there appears to be ectopic expression of sNPF-Gal4 in the intrinsic AKH producing cells. KC, Kenyon cells of the mushroom body. **C1-3**. The three sNPF-expressing neurosecretory cells also display corazonin-immunoreactivity (magenta).

The neurohemal organ of the larval brain, the ring gland, is situated around the anterior aorta (Fig. 5A–C). A variety of brain neurosecretory cells are known to innervate different parts of the ring gland and aorta [27]. We detected varicose axons displaying strong sNPF immunolabel that supply the ring gland and aorta (Fig. 5A–D). Interestingly, it appears that the sNPF-IR axons entering the ring gland extend only into the glandular lobes where the adipokinetic hormone (AKH) producing cells reside [27] (Fig. 5C – E). As seen in Fig. 5D–E it appears that the sNPF-IR axons terminate onto the AKH producing cells visualized with the c929-Gal4 line that displays DIMM expression, characteristic of neurosecretory cells {Park, 2008 #92}. Another supply of sNPF-IR axons terminate on the wall of the anterior aorta (Fig. 4A–C). It was not possible to clearly identify the cell bodies of the neurons (neurosecretory cells) that supply the axons to the ring gland and aorta by ordinary immunolabeling. However, using the sNPF-Gal4 to drive GFP we could show axonal projections to the ring gland and aorta from a set of three LNCs in each hemisphere (Fig. 6A, B). We also used the sNPF-Gal4 driven GFP for double labeling with antiserum to corazonin since this peptide is known to be expressed in three pairs of LNCs in the larva [27-30]. We found that the three pairs of LNCs with projections to the ring gland coexpress sNPF and corazonin (Fig. 6C). Curiously the sNPF-Gal4 expression was also seen in the AKH producing cells of the ring gland, but this is likely to be ectopic expression since we could not detect sNPFp immunoreactivity or *snpf *in situ signal in these glandular cells.

When analyzing the sNPF distribution in the LNCs and ring gland it was also apparent that sNPFp immunoreactive varicose axons terminate close to the dendrites of the insulin-like peptide (ILP) expressing (using a *dilp2*- and c929-Gal4) median neurosecretory cells (Fig 5E, F). This was also seen in a recent paper by Lee et al. [31]. The source of some of these sNPF expressing axons will be described later.

### sNPF in adult brain

#### In situ hybridization

**Figure 7:**
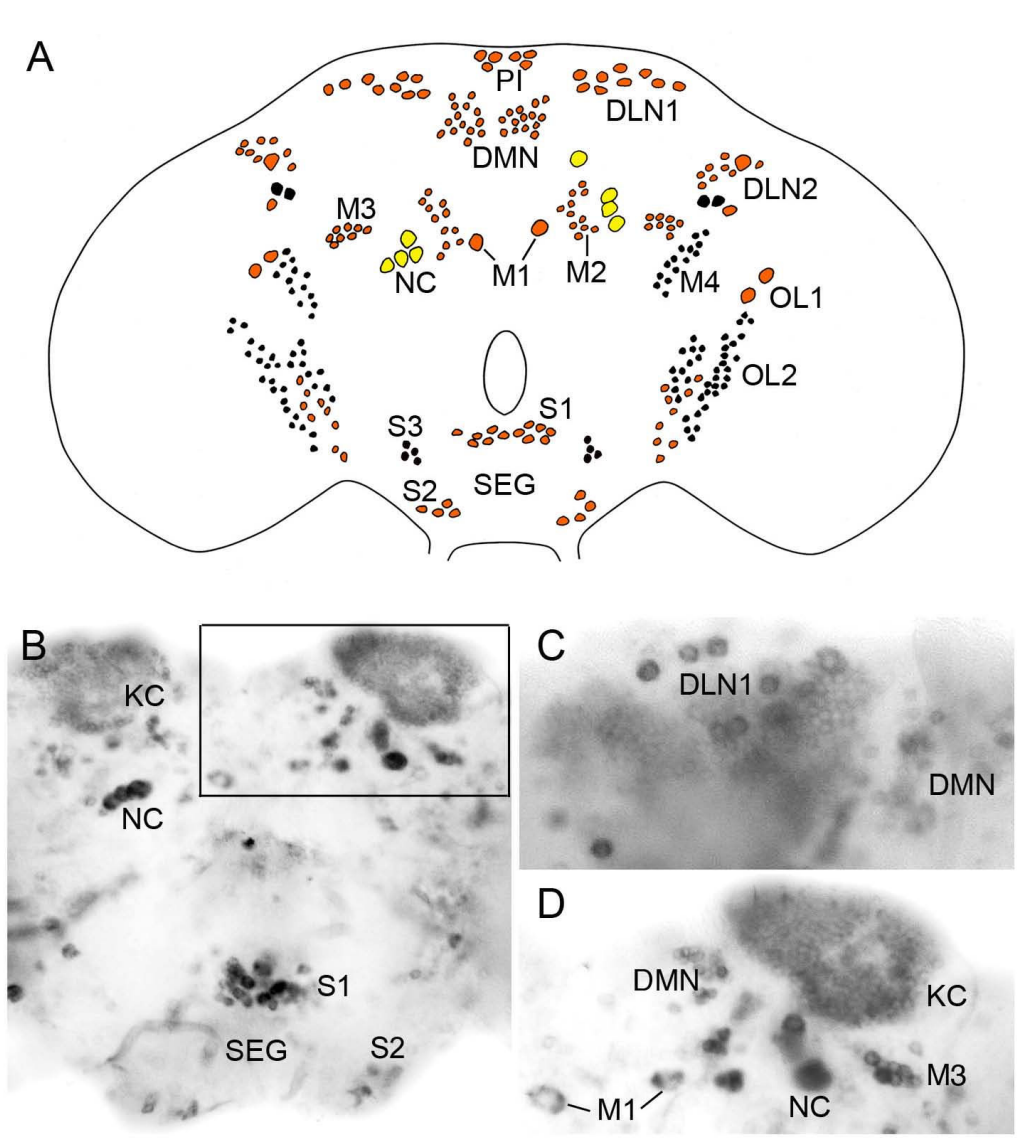
**In situ hybridization with nucleotide probe to *snpf *in the adult brain. The brain and subesophageal ganglion (SEG) is shown in frontal views (dorsal is up). A**. Schematic tracing of *snpf *expressing cell bodies in brain. Red cell bodies are posterior, black ones anterior and yellow are posterior and large intensely labeled ones. The cell body groups in the protocerebrum have been designated as follows: DLN1 and 2, dorso-lateral neurons; PI, pars intercerebralis neurons; DMN, dorsal median neurons, M1 – 4, median neurons. OL1 and 2, optic lobe neurons; NC, possible neurosecretory cells; In subesophageal ganglion: S1 – 3, subesophageal neurons. These designations are used in the following figures for adult neurons. Note that the mushroom body Kenyon cells and numerous small cell bodies distal to the medulla are not shown here. **B**. Micrograph of posterior portion of the brain with in situ signal in Kenyon cells (KC), large, possibly neurosecretory cells, and cell bodies in the subesophageal ganglion (SEG). The framed area is shown in D. Some of the cell bodies from A are indicated. **C**. Detail of cluster of dorsal lateral neurons (DLN1) posterior to the Kenyon cell group (left hemisphere). DMN, small median neurons. **D**. Dorsal brain (right hemisphere) with Kenyon cells (KC), a putative neurosecretory cell (NC), clusters of small cells (DMN and M3) and a pair of median cells (M1).

In situ hybridization with *snpf *antisense probe was performed on wholemounts of adult brains. Additionally to the numerous Kenyon cells (up to 2500 in each mushroom body [26]), a total of at least 280 neuronal cell bodies were seen labeled with the probe in the brain, including the subesophageal ganglion (Fig. 7). Again, three main size classes of neuronal cell bodies were detected and the smallest neurons were the ones displaying variability in intensity of staining and thus the number of clearly detectable cell bodies varied.

In the subesophageal ganglion there is a total of at least 28 neurons distributed in three bilateral groups: one pair of clusters (S1) close to the esophageal foramen, another pair (S2) ventro-laterally in the ganglion, and a third pair (S3) dorso-laterally (Fig. 7A, B). These neurons have cell bodies of small or intermediate size.

At the base of each optic lobe there are clusters of about 50 small cell bodies (OL2), distributed both anteriorly and posteriorly (Fig. 7A). In the dorsal protocerebrum there are several clusters of labeled cell bodies. Most of these are located posteriorly; only four anterior cell bodies were reproducibly labeled (Fig. 7A). The more prominent neuron groups are two dorsal clusters, each with 8 – 10 intermediate sized cell bodies, a set of about 24 small neurons dorso-medially, two pairs of large cell bodies laterally and medially and two sets of four large intensely labeled cell bodies, likely to be neurosecretory (LNCs) (Fig. 7A–D). Also some cell bodies in dorso-lateral protocerebrum display strong *in situ *signals (in the DLN cluster of Fig. 7A). The designations of the neuron groups in Fig. 7A are used in the next section when we describe sNPF-IR neurons.

#### Immunolabeling

**Figure 8:**
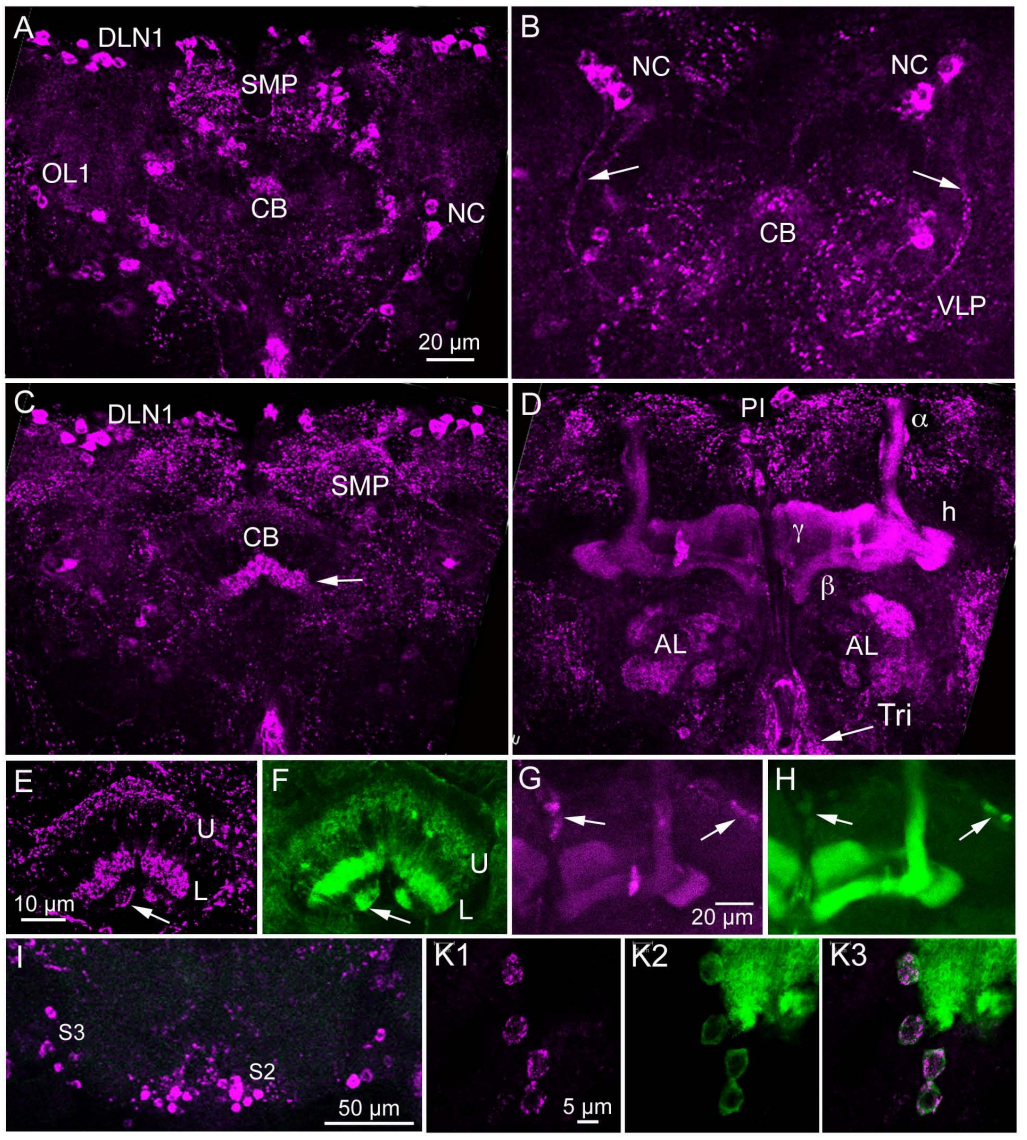
**Distribution of sNPF in the adult brain. **A – D**. Immunolabeling with antiserum to sNPFp in wholemounts of brain. A**. A posterior confocal image with neuronal cell bodies (DLN1, OL1) and two of the four neurosecretory cells (NC). Punctate labeling is seen in superior median protocerebrum (SMP) and in central body (CB; only posterior portion seen here). **B**. More posterior view of another brain with neurosecretory cells (NC) from which axons (arrows) can be seen projecting towards the nerves connecting to the corpora cardiaca. Neuropil labeling is seen in ventrolateral protocerebrum (VLP) and posterior central body (CB). **C**. More anterior optical section with labeling in central body (CB), especially the lower layer (arrow). Other abreviations as in A. **D**. Image at the level of the mushroom body lobes (α, β and γ) and heel (h). Labeling is seen in some glomeruli of the antennal lobes (AL) and in the tritocerebrum (Tri). Two cell bodies are labeled in the pars intercerebralis (PI). **E**. Immunolabeling in the upper (U) and lower (L) layers of the central body (fan-shaped body) and the nodules (arrow). **F**. Expression of sNPF-Gal4-driven GFP in central body for comparison. Note that E and F are from different specimens. **G and H**. Expression of sNPFp immunolabeling (G) and sNPF-Gal4 (H) in the mushroom body lobes (a double labeled specimen) and some cell bodies (arrows). Note the identical expression of markers. **I**. Cell bodies (S2 and S3) in subesophageal ganglion expressing sNPR immunolabeling. **K1 – 3**. A double labeled specimen showing close match between immunolabeling and sNPF-Gal4 expression in four large protocerebral cell bodies close to the calyx of the mushroom bodies (green patch in upper right corner).

Wholemount immunocytochemistry was not optimal for the adult brain. Thus, most data presented here were derived from cryostat sections. Therefore cell body counts were less reliable and were only tried for some neuron groups. In general sNPFp-IR cell bodies were found in the same groups as with in situ hybridization (compare Fig. 7A and 8). Also for the adult brain the smaller cell bodies displayed more variable immunolabeling.

A very large number of neuronal cell bodies were immunolabeled (anti-sNPFp) in the adult brain. As described previously [21] most of the 2500 Kenyon cells of each of the mushroom bodies were weakly labeled. Approximately the same numbers of cell bodies were seen in proto-, deuto- and tritocerebrum and in the subesophageal ganglion as seen after *in situ *hybridization. Most of the cell bodies were small, but some larger and intermediate sized ones were also detected. Two clusters of larger cell bodies were seen posteriorly in the protocerebrum (four in each hemisphere) and are likely to be neurosecretory cells (Fig. 8A, B; yellow ones in Fig 7A). The prominent dorsal protocerebral cell groups, DLN1, were intensely immunolabeled (Fig. 8A, C). These DLN1s may include small LNCs known to express corazonin (see Fig. 6C) [28,29].

Most of the neuropil regions of the brain were supplied by varicose sNPFp-IR processes. In the protocerebrum punctate immunolabeling was seen in two layers of the fan-shaped body of the central complex (Fig. 8C, E), in the lobes of the mushroom bodies (Fig. 8D), in the basal portion of the medulla and in the lobula of the optic lobe (see later section; Fig. 10) and diffusely in non-glomerular neuropil throughout the protocerebrum (Fig. 8A–D). No labeling was detected in the ellipsoid body or the protocerebral bridge of the central complex (not shown).

In the deutocerebrum punctate immunolabeling was seen in a subset of glomeruli of the antennal lobes (Fig. 8D). This immunolabeling is located in the olfactory receptor neurons (ORNs) of the antenna and their axons and will be described in more detail in conjunction with an acetylcholine marker (*Cha*-Gal4) in a later section (referring to Fig. 13A–C). Some cell bodies were found adjacent to the antennal lobe, but we could not resolve any processes entering the glomeruli suggesting that none of the local and projection interneurons of the antennal lobe express sNPF. Tritocerebral neuropil contains a dense supply of sNPFp-IR processes (Fig. 8D).

Extensive immunolabeling was seen in processes in subesophageal neuropil regions and in the three different clusters of cell bodies indicated by in situ hybridization (Fig 8I). However, there was no evidence for afferent axons of sensory neurons (such as gustatory receptors).

### Expression of sNPF-Gal4 in the adult brain

**Figure 9:**
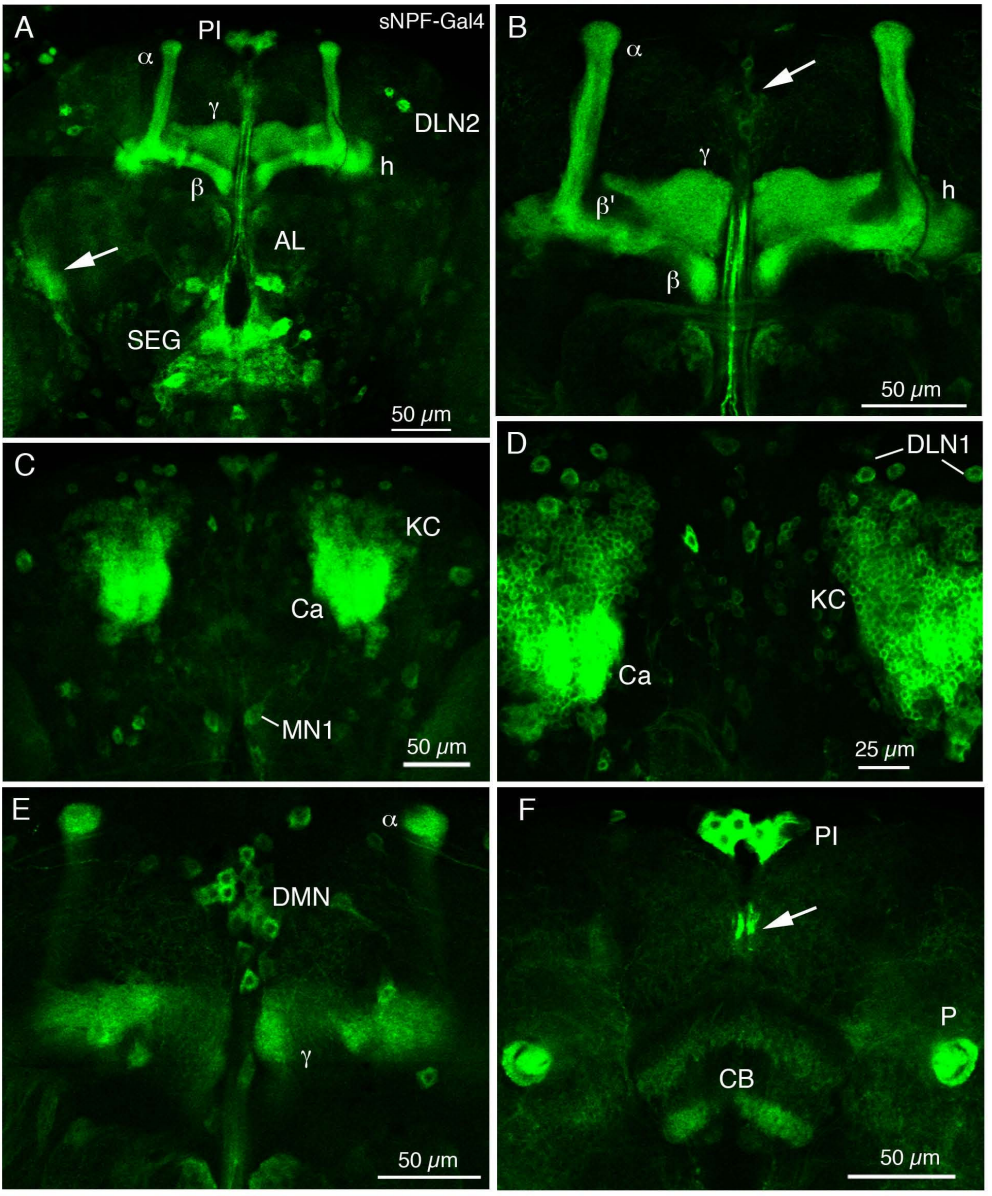
**Distribution of sNPF-Gal4 driven GFP expression in adult brain. The Gal4 line NP6301 drives GFP in neurons that largely correspond to the sNPFp-IR neurons in the adult brain.A**. Overview of brain in confocal section at level of mushroom body lobes. Strong expression is seen in axons in the α, β and γ-lobes and heel (h) of the mushroom body, and in processes in neuropil of the subesophageal ganglion (SEG). In glomeruli of the antennal lobe (AL) labeling is weaker at this level. Several groups of neuronal cell bodies are seen (e. g. PI and DLN2). A neuropil at the base of the lobula (arrow) is strongly expressing sNPF-Gal4 (see Fig. 10 for details). **B**. Detail of mushroom body lobes. The α, β and γ-lobes and heel (h) contain fibers expressing sNPF, whereas the α' (not seen) and β' do not. The arrow indicates a set of small median neurons (DMN in E). **C**. Overview of dorsal brain at level of mushroom body calyces (Ca). Kenyon cells (KC) and their processes express sNPF. A pair of large median neurons (MN1, see Fig. 7A) can be seen. **D**. Detail of mushroom body calyces (Ca) with numerous Kenyon cells (KC) and sets of DNL1 neurons. **E**. A set of dorsal median neuron cell bodies (DMN) located between the tips of the α-lobes. **F**. Cell bodies of the median neurons in the pars intercerebralis (PI) and their axons (arrow), dorsal to the central body (CB). Two layers of sNPF-expresing processes are seen in the central body. Strong sNPF expression is seen in axons of the cross-sectioned mushroom body peduncles (P).

In the adult brain the sNPF-Gal4 expression was most obvious in the mushroom bodies, but also in most other neurons indicated from immunocytochemistry and in situ hybridization (Fig. 9). Most of the neuronal cell groups shown in Fig. 7 could be identified (examples in Fig. 9). Gal4 expression was seen in detail in neuronal processes in the mushroom body calyx and lobes (Fig. 8H, 9A, B), Kenyon cells (Fig. 9C, D), the fan-shaped body of the central complex (Fig. 8F, 9F), and in neuropils of the subesophageal ganglion (Fig. 9A). The expression in antennal lobe glomeruli was weaker (Fig. 9A).

Double labeling with Gal4-GFP expression and anti sNPFp was performed to document colocalized markers (examples in Fig. 8E-H, K). In wholemounts only more superficially localized cell bodies and neuropils can be readily visualized with the antiserum; thus we do not have a complete map. However, the match between markers is fairly good in that sNPF-IR material was rarely seen without GFP; conversely there was likely ectopic expression of sNPF-Gal4 in some neurons (including axons of R7 and 8 from the retina). In summary, the Gal4 expression is not likely to reflect the sNPF expression faithfully, but is good enough to give an overall view of the diversity of sNPF neurons. Also, in neurons that can be confirmed by sNPFp antiserum the expression is very useful for revealing neuron morphologies since the immunolabeling is too punctate for that.

### Expression of sNPF-Gal4 and immunolabeling in the optic lobe

**Figure 10:**
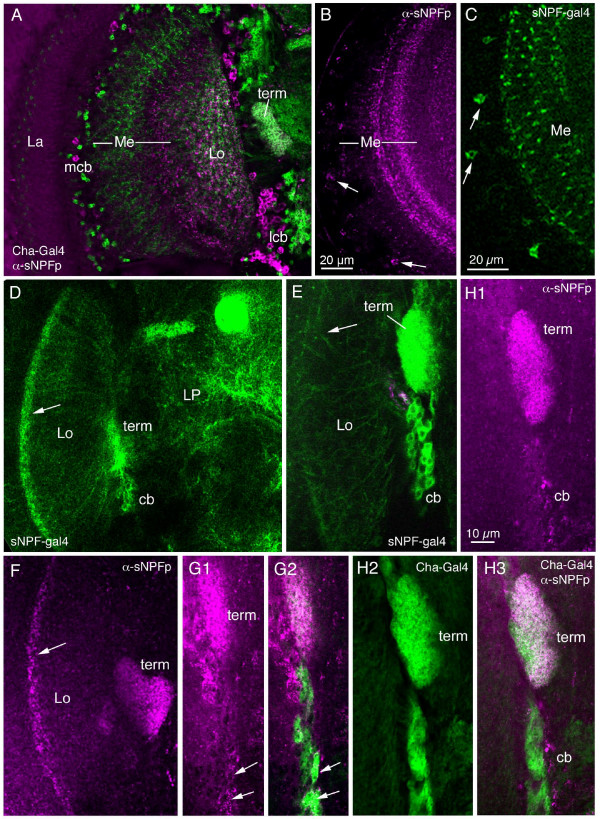
**Expression of sNPF in the optic lobe. The left optic lobe is shown in frontal views (dorsal is up, the retina is to the left).A**. Overview of optic lobe with anti-sNPF precursor (α-sNPFp) in magenta and Cha-Gal4-driven GFP (green) as a reference marker. Cell bodies expressing sNPFp are seen (mcb), mixed with Cha-expressing ones, distal to the medulla (Me), and at the base of the lobula (Lo), lcb. Immunolabeling in neuropils is seen in medulla and lobula, but not in the lamina (La). Also a neuropil basal to the lobula express sNPFp and Cha (term). **B**. Detail of sNPFp expression in medulla. Note two prominent layers of immunoreactivity in the basal medulla and scattered cell bodies distal to the neuropil (arrows). **C**. Expression of sNPF-Gal4 in the medulla (slightly oblique optical section). In addition to distal cell bodies (arrows) and neuropil processes basally, labeling is seen in R7 and R8 photoreceptor axons (likely to be ectopic Gal4 expression). **D**. Overview of sNPF-Gal4 expression in lobula and lateral protocerebrum (LP). In the lobula a distal layer (arrow) strongly expresses GFP. One population of columnar lobula neurons appear to terminate in a distinct neuropil (term) and have their cell bodies (cb) in a cluster at the base (part of the OL2 in Fig. 7A). **E**. Detail of columnar lobula neurons with cell bodies (cb) and terminations (term). These neurons have presumed dendrites distally in the lobula (arrow). Compare to immunolabeling in 10F and 10H1. **F**. Immunolabeling with α-sNPFp reveals a pattern similar to the sNPF-Gal4 in D.**G1 and G2**. Some of the sNPFp-IR cell bodies (arrows) of the lobula cluster coexpress Cha-Gal4 (G2). Term, neuropil with terminations of sNPFp expressing neurons. **H1 – H3**. Other sNPFp-IR cell bodies (cb) of the lobula cluster do not coexpress Cha-Gal4.

Except for the sNPF-Gal4 expression in the R7/8 photoreceptors there is a good match with sNPFp immunolabeling in the optic lobe of the fly. We found sNPFp immunoreactive neurons in the medulla and lobula, but not lamina and lobula plate (Fig. 10A, B). In the medulla there are sNPFp-IR cell bodies scattered distal to the neuropil and two dense layers of varicose processes in the basal neuropil (Fig. 10B). The Gal4 expression is similar with respect to cell bodies and the basal layers (Fig. 10C). In the lobula sNPF-Gal4 expression was seen in a set of columnar neurons with cell bodies at the ventral base of the neuropil and a termination area in the lateral protocerebrum (Fig. 10D, E). Especially in the distal lobula the expression is strong. The sNPFp antiserum labels a similar pattern in the lobula (Fig. 10F, G) although the resolution of processes is far better with the Gal4 driven GFP. We used a *Cha*-Gal4 driver to display optic lobe neurons likely to express choline acetyltransferase (Cha) and thus be cholinergic (Fig. 10A, F, H). Most of the *Cha*-expressing neurons do not colocalize sNPFp immunolabel but there is *Cha*-Gal4 expression in a set of columnar neurons similar to the sNPF-expressing ones (compare Fig. 10E and 10F). Both sNPF and *Cha *markers reveal the dense termination site in the lateral protocerebrum and cell bodies in a similar position. It appears as if a small subset of the Cha-expressing neurons colocalize sNPF (Fig. 10H).

### Expression of DIMM and sNPF

**Figure 11:**
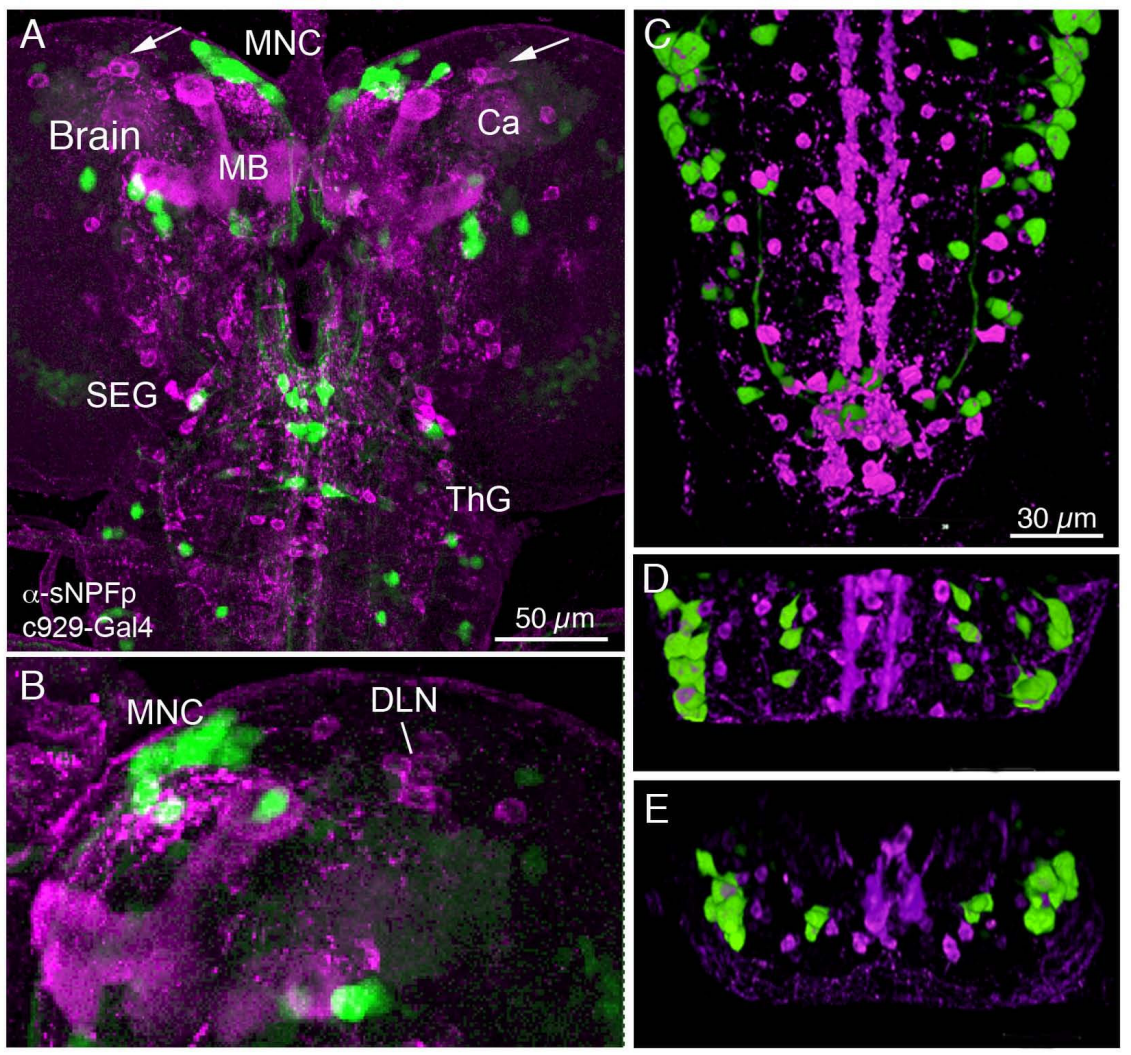
**Larval sNPFp immunoreactive neurons do not coexpress DIMM. Double labeling of larval CNS with antiserum to sNPFp (magenta) and *dimm*-Gal4-driven GFP (c929-Gal4), voltex projections.A**. Brain, subesophageal ganglion (SEG) and thoracic ganglia (ThG) with *dimm*-expressing neurons distinct from sNPFp-expressing ones. In the DLN cluster (arrows) in the brain corazonin-immunoreactive neurons (see Fig. 6C) can be found. These sNPF-corazonin expressing neurons could possibly coexpress *dimm *[15] (but GFP may be too weak here to be seen). MNC, median neurosecretory cells; MB, mushroom body lobes; Ca, calyx. **B**. Higher magnification of right brain hemisphere of another larva with the sNPFp-expressing DLN cluster of cell bodies and *dimm*-expressing median neurosecretory cells (MNC). No *dimm*-expression is seen in any of these neurons. **C – D**. Double labeling with anti-sNPFp and c929-Gal4 in larval ventral nerve cord shown in maximum projections and voltex reconstruction. No colocalization of markers is seen. **C**. Ventral view of the segments A3 – A9. **D**. Dorsal view of the segments A1 – A2. **E**. Cross section (rear view) of the segments A1 – A2.

The transcription factor DIMMED (DIMM) is known to specify a functional phenotype in a large subset of peptidergic neurons [15,24]. These DIMM-expressing neurons, identified by the c929-Gal4 line, are larger secretory cells and rarely small peptidergic interneurons [32]. We used the c929-gal4 to drive GFP for a comparison with sNPFp-immunolabeling in third instar larvae. As shown in Fig. 11, we detected no colocalization of markers in any of the neuronal cell bodies of the brain (Fig. 10A, B) or ventral ganglia (Fig. 10C–E). It cannot be excluded that some brain neurons displayed too weak GFP expression to be detected here.

### Many neurons expressing sNPF may colocalize other neurotransmitters

Since a large number of smaller neurons express sNPF in *Drosophila *we found it likely that some would colocalize other neuroactive compounds, such as classical neurotransmitters, monoamines of even other neuropeptides. We thus used several different Gal4 lines to drive GFP to display neurons likely to produce dopamine (*th*; tyrosine hydroxylase), octopamine/tyramine (*tdc*; tyrosine decarboxylase) acetylcholine (*Cha*; choline acetyltransferase), glutamate (OK371; vesicular glutamate transporter; vGluT), GABA (*Gad1*; glutamic acid decarboxylase) and NPF (dNPF; long NPF). We also combined sNPF-Gal4 driven GFP with antisera to corazonin and ILP7.

#### Cholinergic neurons

**Figure 12:**
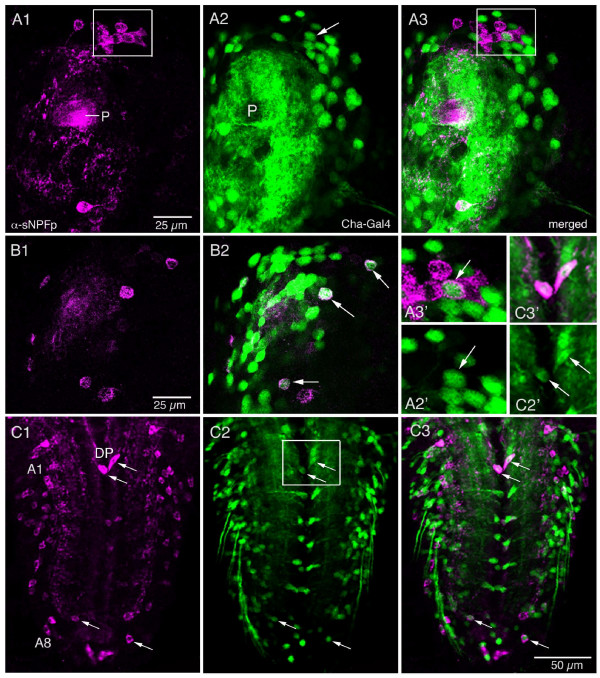
**Distribution of sNPF in relation to putative cholinergic neurons in the larval CNS.** Antiserum to sNPFp (magenta) and *Cha*-Gal4-driven GFP (green) were used as markers. Single confocal sections are shown (anterior is upwards). **A1 – 3**. In the brain (protocerebrum) there are a few cell bodies in a dorso-lateral cluster displaying colocalized markers: one (arrowed in A2) is seen in boxed area of A3. This is also shown in enlarged view in **A2' **and **A3'**. **B1 – 2**. More dorsally in the brain a few other cell bodies in the protocerebrum display colocalized markers (arrows). **C1 – 3**. In the ventral nerve cord (abdominal ganglia) colocalized markers were seen in a small number of cell bodies (arrows). The boxed area (shown in higher magnification in C2' and C3') contains a pair of dorsal neurons (DP) in A1 that colocalize *Cha *and sNPFp. A few more posterior abdominal neurons with colocalized markers were found in adjacent sections.

*Cha*-Gal4 expression, diagnostic of acetylcholine, is known to be abundant in numerous neurons of the *Drosophila *CNS [33,34]. Thus it is not surprising that *Cha*-Gal4 expression is seen in many of the sNPF-expressing neurons. In the larval CNS we detected colocalization of *Cha*-Gal4 and sNPF in subsets of neurons in the DLN cluster of the protocerebrum (Fig. 12A) and in single neurons scattered dorsally in the brain (Fig. 12B). In the ventral nerve cord of larvae some of the intermediate sized cell bodies coexpressed *Cha*-Gal4 and sNPF: the dorso-median pair (DP) in A1 (Fig. 12C) and some of the lateral neurons in A7 and A8 (Fig. 12C).

**Figure 13:**
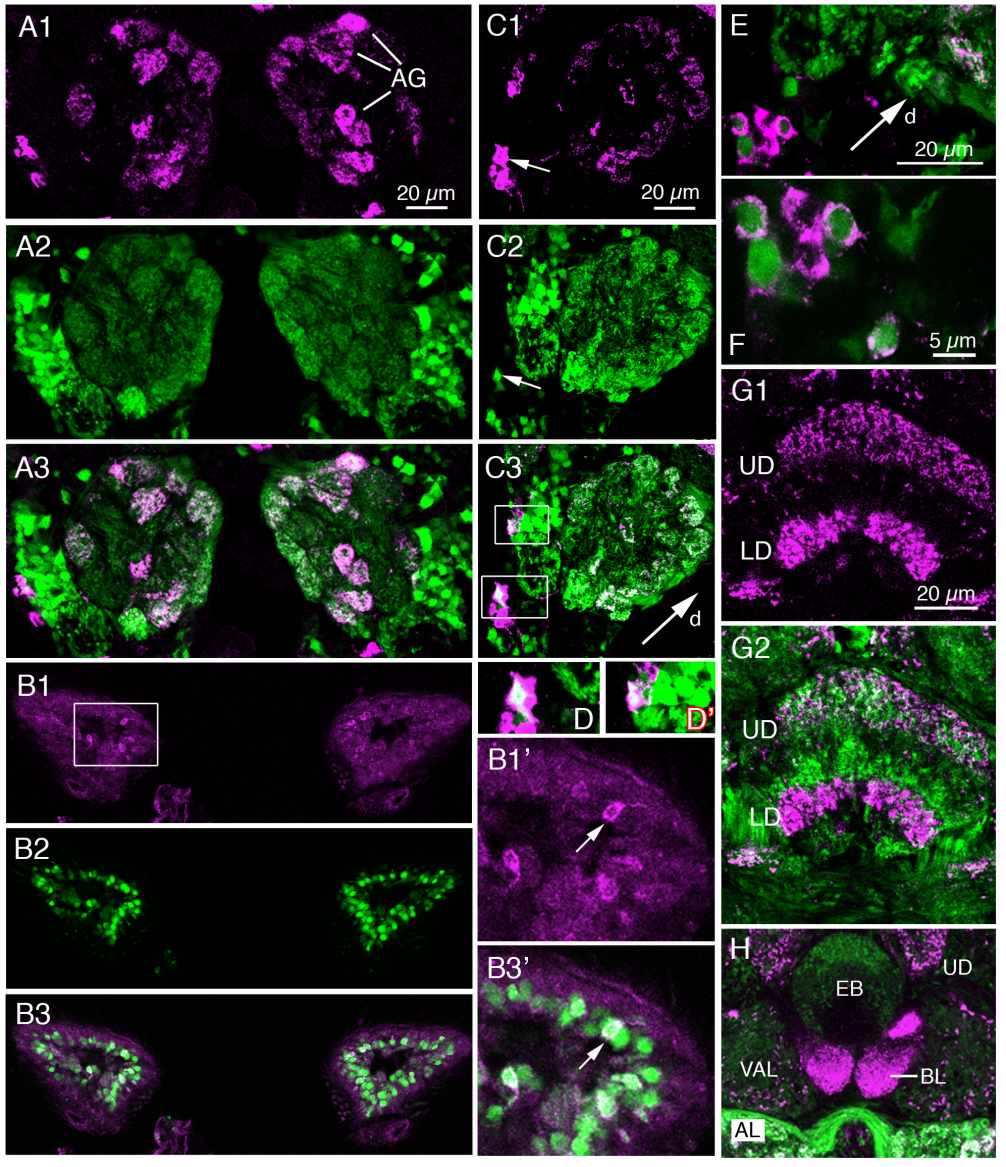
**Distribution of sNPF in relation to putative cholinergic neurons in the adult brain.** Brain is shown in frontal sections (dorsal is up). Antiserum to sNPFp (magenta) and *Cha*-Gal4-driven GFP (Green) revealed some neurons where markers are colocalized (note that the P{Cha-GAL4.W}19B is a fusion with cytoplasmic GFP). **A1 – 3**. Distribution of sNPF in axon terminations of olfactory receptor neurons (ORNs) in a subset of antennal glomeruli (AG). The *Cha *expression is seen in both ORNs and projection neurons of the antennal lobe. **B1 – 3**. sNPF immunolabeling in cell bodies of a subset of Cha expressing ORNs in the antennae. The boxed area in B1 is shown magnified in B1' and B3'. The arrows in B1' and B3' indicate one of the ORNs expressing both markers **C1 – 3**. *Cha *and sNPF is also colocalized in interneurons in the deutocerebrum and subesophageal ganglion (arrows and boxed areas). Note that the GFP is mainly nuclear here. These images are slightly tilted; dorsal (d) is in direction of arrow. The boxed areas are shown enlarged in D and D'. **E and F**. Colocalization of *Cha*-expression and sNPF immunolabeling in a set of small cells laterally in the subesophageal ganglion (S3 neurons in Fig. 7A). **G1 – 2**. In the central body (fan-shaped body shown here) there is no neuronal colocalization of *Cha *and sNPF markers. The sNPFp antiserum labels neuronal processes in two layers (UD and LD) of the fan-shaped body, whereas *Cha*-expression is seen other processes in the UD and in a layer between this and the LD. **H**. There is no sNPFp-immunolabeling in the ellipsoid body (EB) of the central complex, whereas *Cha *expression is seen. VAL, ventral accessory lobe; BL, beta-lobe of mushroom body; AL, antennal lobe. The magnification of H is the same as in A.

In the adult fly we found that a set of the sNPFp immunoreactive cell bodies of ORNs in the antennae (Fig. 13B) and maxillary palps (not shown) and their terminations in the antennal glomeruli coexpress *Cha*-Gal4 (Fig. 13A). Whereas all antennal glomeruli display *Cha*-expressing processes, only a smaller set expresses sNPF immunolabel (Fig. 13A). We also used the Or83b-Gal4 to drive GFP in most of the ORNs of the antennae [35] for labeling with sNPFp antiserum and revealed a similar colocalization pattern (not shown). Antennal lobe projection interneurons express *Cha*, but not sNPF (Fig. 13A). Several interneurons in the proto- and deutocerebrum as well as the subesophageal ganglion also coexpress the two markers (Fig. 13C–F). Most of these interneurons could not be individually identified. It appears as if there is no colocalization of markers for *Cha *and sNPF in neuronal processes in the central complex; the two markers were seen in different layers of these neuropils (Fig. 13G, H). We also found no colocalization in other glomerular neuropils including mushroom bodies. Probably colocalized markers are mostly confined to neuronal processes in non-glomerular neuropils (and in ORN processes in antennal glomeruli).

#### GABAergic neurons

**Figure 14:**
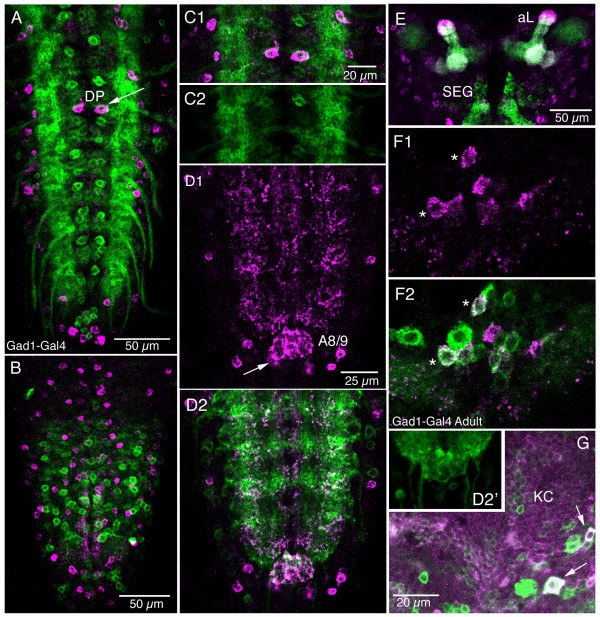
**Double labeling with antiserum to sNPFp and *Gad1*-Gal4-driven GFP.** Single confocal sections (horizontal views; anterior to top) are shown. **A – C**. No colocalization of the markers were seen in neurons of the larval ventral nerve cord (**A **dorsal aspect, **B **ventral aspect). The dorsal DP neurons in first abdominal ganglion (arrow in A) did not colocalize markers, although similar neurons expressing *Gad1*-Gal4 were seen in adjacent neuromeres. In **C1-2 **details of A1 are shown. **D1 – 2**. The sNPF-IR processes in neuropil overlap *Gad1*-Gal4 expressing branches in lateral neuropil of abdominal ganglia. Also the posterior neuropil (terminal plexus) in A8/9 (arrow) expresses both markers but, without colocalization (see also **D2'**). **E**. In the larval brain no colocalized markers were detected in neuronal cell bodies. The colocalization of Gad1-Gal4 and sNPF in the mushroom body lobes (aL) may be irrelevant here, since the Gad1-Gal4 does not support a GABAergic phenotype (see [85]). Most Gad1 expressing cell bodies are in the subesophageal ganglion (SEG) in this optical section. **F1 – 2**. In the adult brain colocalized markers (asterisks) can be seen in a group of dorsal median neurons in the protocerebrum (in the DMN cluster in Fig. 7A). **G**. In the dorso-lateral protocerebrum at the base of the Kenyon cell cluster (KC) some neurons (arrows) colocalize sNPF and *Gad1*-Gal4 (in the DLN2 cluster in Fig. 7A).

Putative GABAergic neurons were revealed by *Gad1*-Gal4 expression. No colocalization of sNPFp and Gad1-Gal4 expression was detected in neuronal cell bodies of the larval CNS (Fig. 14). Substantial superposition of labeled processes was, however, detected (Fig. 14D). In the adult brain few neurons were found to colocalize Gad1 and sNPF markers, in the DMN and DLN2 clusters (Fig. 14F, G; compare Fig. 7A for location).

#### Glutamatergic neurons

**Figure 15:**
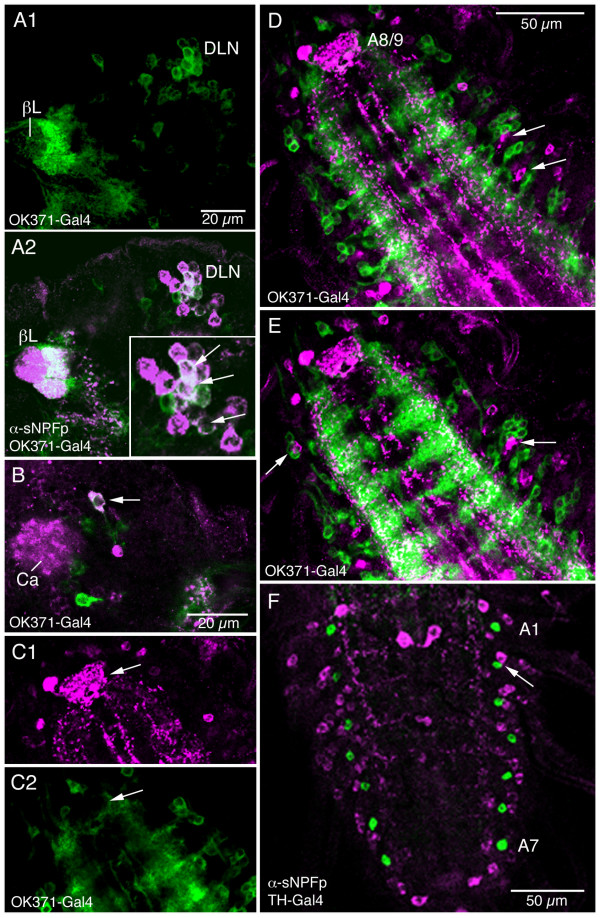
**Labeling with antiserum to sNPFp and OK371- or *TH*-Gal4-driven GFP in the larval CNS.** These Gal4 lines display expression of vesicular glutamate transporter (vGluT) and tyrosin hydroxylase, respectively. **A1 – A2**. Some colocalization of vGluT and sNPF was seen in a cell body group in the dorso-lateral protocerebrum (DLN; see Fig. 1E). A magnified view is shown in inset (arrows indicate cell bodies with colocalized markers). The beta-lobe (βL) of mushroom body is surrounded by OK371-expressing processes. **B**. More dorsally in the brain another cell body co-expresses markers (arrow). Ca, calyx of mushroom body. **C – E**. In the ventral nerve cord (abdominal ganglia shown here; anterior downwards) vGluT and sNPF appear not to be colocalized. In **C1 **and **C2 **sNPF-IR processes are seen in a dense terminal plexus neuropil of A8/9 (arrow), that is not supplied by vGluT expressing fibers. In **D **and **E **it can be seen that sNPF-IR cell bodies are located in clusters with vGluT expressing ones (arrows). In the more dorsal focal plane in E it is apparent that varicose sNPF-IR fibers run through neuropils with dense supply of vGluT expressing processes. **F**. Tyrosin hydroxylase expression (TH-Gal4) is seen in a pair of cell bodies in each of the abdominal ganglia A1 – A7 (green). These cell bodies are clustered with sNPF-IR cell bodies (e. g. at arrow), but no colocalization is seen anywhere in the CNS.

We revealed putative glutamatergic neurons in the larval CNS with the OK371-Gal4 driver, known to reveal vGluT distribution [36]. Some neurons in DLN cluster and others near the mushroom body calyx of the larval protocerebrum colocalize OK371-Gal4 and sNPF expression (Fig. 15A, B). No colocalization was seen in larval ventral nerve cord, although superimposed neuronal processes were detected in lateral neuropils (Fig. 15E-C).

#### Dopaminergic neurons

Tyrosine hydroxylase (*th*-Gal4) expression correlates well with dopamine producing neurons [37,38]. We could not detect any colocalization of *th*-Gal4 and sNPF expression in neuronal cell bodies of the larval or adult CNS (Fig. 15F; see also [21]). However the *th*-expressing neurons of the abdominal ganglia cluster with sNPF-IR neurons laterally in the neuromeres (Fig. 15F). No colocalization was seen with tyrosine decarboxylase-driven GFP in the larval CNS (not shown; but see [21]).

#### Peptidergic neurons

**Figure 16:**
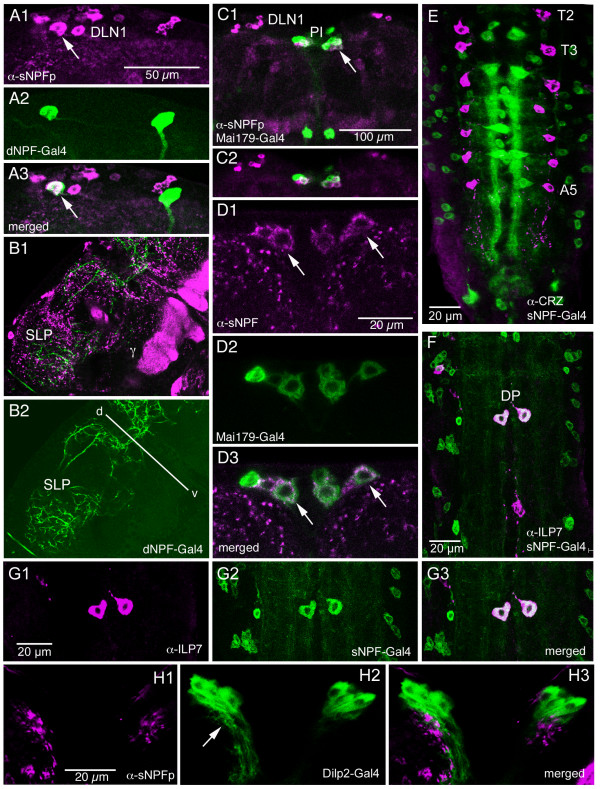
**Relation between sNPF-expression and other neuropeptides in the CNS.** Labeling with antiserum to sNPFp and GFP driven by Gal4s displaying peptide expression (dNPF, Mai179 and Dilp2) or antiserum to insulin-like peptide 7 (ILP7). **A1 – 3**. In the adult brain colocalization of sNPF-immunolabeling and dNPF-Gal4 expression (long NPF) is seen only in one cell body per hemisphere (arrow). These cell bodies are located in the DLN1 cluster of the dorsal protocerebrum. **B1 – 2**. In the adult dorsal protocerebrum (SLP) there is a dense supply of neuronal processes expressing sNPF (magenta) and dNPF (green), but no colocalized markers are seen in these processes. These images are slightly tilted (the dorso-ventral axis is indicated by the d-v line). γ, gamma lobe of mushroom body. **C1 – 2**. The Mai179-Gal4 drives GFP in a specific set of neurons (PI) in the adult pars intercerebralis (arrow). In a subset of these we find sNPF colocalized. DLN1, dorsal lateral neurons. **D1 – 3 **Details of Mai179-Gal4 and sNPF expression in adult pars intercerebralis neurons. At least four of these neurons express both markers (e. g. at arrows). **E**. In the larval thoracic-abdominal ganglia the neuropeptide corazonin (CRZ) is expressed in neurons (magenta) distinct from the sNPF-Gal4 expressing ones (green). **F**. Insulin-like peptide 7 (ILP7) immunoreactivity is detected in the DP neurons in A1 that co-express sNPF-Gal4 (merged image stack). **G1 – 3**. Single sections showing the coexpression of ILP7 and sNPF in DP neurons. **H1 – 3**. Relation between sNPFp-immunolabled axon terminations (magenta) and dendrites (arrow) of Dilp2-Gal4 expressing neurons (green) in pars intercerebralis. Note that the sNPF-expressing terminations impinge on the dendrites. Some of the sNPF-IR processes seen here are likely to be derived from the DP neurons.

We used several markers for peptides to reveal relations to sNPF-expressing neurons (Fig. 16). First, we were interested to reveal whether the sNPF antiserum cross reacted with dNPF (long NPF). Analyzing distribution of GFP driven by a dNPF-Gal4, known to be specific for dNPF expression[39], with antiserum to sNPFp, we found that only two neurons in the adult brain colocalize markers (Fig. 16A). These are in the DLN1 cluster. There is extensive superposition of sNPF and dNPF-labeled processes in the dorsal protocerebrum. In the larval CNS there is no colocalization at all (see [21]).

The Mai179 enhancer trap line drives GFP in subsets of brain neurosecretory cells and other, possibly peptidergic, neurons [27]. We found that a small set of Mai179 expressing neurons (PI) in the adult pars intercerebralis coexpress sNPF (Fig. 16C, D). These PI neurons appear to be interneurons rather than neurosecretory cells.

Corazonin is expressed in a small set of neurons and neurosecretory cells in the *Drosophila *CNS [29,30]. We have shown already that three pairs of brain neurosecretory cells colocalize sNPF and corazonin (Fig. 16C). In the ventral nerve cord there is a set of 14 segmentally distributed corazonin-immunoreactive neurons. These do not colocalize sNPF (Fig. 16E).

It has been shown that insulin-like peptide 7 (ILP7), with resemblance to mammalian relaxin, is distributed in interneurons and efferent neurons of the *Drosophila *abdominal ganglia [40-42]. One pair of ILP7 expressing neurons was described dorsally in A1. We applied antiserum to ILP7 to flies bearing sNPF-Gal4 and UAS-GFP transgenes and found that indeed the sNPF expressing DP neurons of A1 co-express ILP7-immunolabeling (Fig. 16F, G). No other neurons coexpress the two markers in the larval CNS.

Since it was shown that the ILP7-expressing DP neurons have ascending axons that terminate on the dendrites of the ILP producing median neurosecretory cells (MNCs) of the larval brain [41] we wanted to analyze sNPF immunolabeling in relation to these MNCs. We used a Dilp2-Gal4 line to identify the ILP producing MNCs in the larva and found that indeed sNPFp-immunolabeled axon terminations localize close to the MNC dendrites in the pars intercerebralis (Fig. 16H). Using the sNPF-Gal4 and antiserum to ILP7 we also showed that the ILP7 expressing axon terminations in the brain coexpress sNPF (not shown).

The main types of sNPF neurons co-expressing other markers for chemical signaling components are summarized in Table 1.

**Table 1:** Neuronal colocalization of sNPF with other neuroactive substances

Neuron type	colocalized substance	acronym	location	stage	figure
LNCs^1^	corazonin	LNCs (DLG)	brain	larva^2^	6C
ORNs	acetylcholine (Cha)		antenna	adult	13A, B
interneurons	Cha	DLG DP, others	brain v. gangl	larva	12A – C
interneurons	Cha	S3, others	brain^3^	adult	13C, F, 10G
interneurons	neuropeptide F	DLN1	brain	adult^4^	14A
interneurons	ILP-7	DP	v. gangl	larva^2^	14F, G
interneurons	GABA (GAD1)	DMN, others	brain	adult^4^	Add. M. 3
interneurons	glutamate (vGluT)	DLN, others	brain	larva^2^	Add. M. 4

^1 ^Neurosecretory cells

^2 ^Adult not analyzed

^3 ^Also in lobula of optic lobe

^4 ^No colocalization detected in larvae

### Mass spectrometry of *Drosophila *CNS reveals sNPF-1

There is some ambiguity with respect to which of the *snpf*-derived peptides that are expressed in the CNS of *Drosophila*. In several published accounts only sNPF-1 and sNPF-1_4–11 _(sequence identical to sNPF-2_12–19_) have been clearly revealed by mass spectrometry of larval CNS extract or dissected neurohemal organs [43-45], whereas in one paper [46] masses indicating also sNPF-2, 3 and 4 were detected in HPLC-purified whole body extracts. The identity of the peptides behind these masses were, however, not confirmed with fragmentation analysis. Here we used single microdissected portions of the larval and adult brain for mass spectral analysis by direct peptide profiling. These portions included the mushroom bodies, but neither the subesophageal ganglion, optic lobes (or the optic lobe anlagen in larvae), nor the larval ring gland or adult neurohemal organs. In the larval brain only sNPF-1_4–11 _and sNPF-2_12–19 _were detected (Additional File 3A). In the adult brain portion we detected a major peak with the mass corresponding to sNPF-1_4–11 _and sNPF-2_12–19_, but only a very small one corresponding to sNPF-1 (Additional File 3B, C). Fragmentation analysis confirmed the major mass peak as sNPF-1_4–11 _(Additional File 3D). No trace of full length sNPF-2, sNPF-3 or sNPF-4 was detected in brains of either developmental stage. In congruence with the present mass spectrometric data only sNPF-1_4–11_/sNPF-2_12–19 _has so far been isolated biochemically and sequenced from *Drosophila *[21]. In the larval ring gland a more substantial peak representing sNPF-1 was detected, but the sNPF-1_4–11_/sNPF-2_12–19 _signal was far stronger [44]. Thus, it appears that except for sNPF-1, the originally predicted peptides of the sNPF precursor are not liberated at detectable levels; only the octapeptide SPSLRLRFamide can be detected at high levels in the CNS.

## Discussion

### A diversity of neuron types express sNPF

We have investigated the widespread neuronal expression of a *Drosophila *neuropeptide gene *snpf *and its peptide products, sNPFs, in the larval and adult nervous system of the fruitfly. This mapping was made by in situ hybridization, a *snpf*-Gal4 line and immunocytochemistry with antiserum specific for the *snpf*-derived precursor protein. A large population of diverse neurons types was revealed with the different sNPF/*snpf *markers: a few neurosecretory cells, numerous interneurons of many kinds as well as olfactory receptor neurons (ORNs) of the antennae. This wide expression of sNPFs in diverse neuron types, many of which may colocalize different neurotransmitters, suggests a diversity of functions beyond that of any insect neuropeptide studied so far. Especially the finding of numerous small neurons such as Kenyon cells of the mushroom bodies and olfactory receptor neurons (ORNs) of the antennae and small neurons of the optic lobe suggest that sNPFs play important roles in local cotransmission and/or neuromodulation.

Since the intensity of immunolabeling with the sNPFp antiserum was variable, especially for the smaller neuronal cell bodies, it was hard to provide definite numbers of smaller sNPF expressing neurons, in spite of analyzing many specimens. Possibly part of this variability in immunolabeling reflects *bona fide *fluctuations in sNPF levels related to activity levels of neurons. Another drawback with the immunolabeling was that it was mostly punctate within neuronal processes and thus did not allow resolution of neuronal morphology beyond location of cell bodies, major tracts and axon terminations. Therefore, the majority of the sNPF expressing neuron types remain anonymous cell bodies that cannot be connected to their release sites. Apart from ORNs, a prominent exception to the anonymity is seen for sNPF expression in the numerous Kenyon cells that send sNPF-IR processes into the α, β and γ lobes, but not the α' and β' lobes and α_c_β_c _portions of these lobes [21]. Two other sets of neurons that could be revealed in detail are the unique pair of DP neurons in the first abdominal neuromere (A1) in larvae that co-express ILP7 and the lateral protocerebral cells (LNCs) coexpressing corazonin.

One aim here was to obtain data that could reveal functional roles of sNPF expressing neurons. Does sNPF(s) have distinct functions or is the function dependent on the context of the neurons releasing the peptide(s). Generally neuropeptides are diverse and multifunctional signal substances [3,14,47-50]. In insects more than 30 genes encode precursors of neuropeptides that are known to regulate many aspects of development, growth, reproduction, metabolism, homeostasis and behavior [3,14,49-54]. Most of these regulatory roles are played by neuropeptides released as hormones into the circulation and some may act after episodic bulk release within the CNS [15,55]. In addition, neuropeptides in interneurons are likely to act as local cotransmitters or neuromodulators in central neural circuits [16,48]. Peptidergic neurons thus are of several different major types [16,15]: (1) large neurosecretory cells with peripheral release sites that produce hormonal peptides, (2) a variety of circuit interneurons, some of which are relatively small, produce peptidergic cotransmitters (or local neuromodulators), (3) a separate type of peptidergic interneurons of intermediate to large size are likely to be responsible for episodic bulk transmission within the CNS and (4) subsets of motoneurons are known to express the peptide proctolin as cotransmitters [1,17,18]. To these types of peptide expressing neuron types we can now add sNPF expressing chemosensory neurons (ORNs) of the *Drosophila *antennae and maxillary palps.

A recent paper [15] addressed the role of the transcription factor DIMM in determining functional phenotypes of peptidergic neurons. Expression of DIMM was seen only in a portion of the known peptidergic neurons of *Drosophila*, namely a set referred to as Large cells that Episodically release Amidated Peptides (LEAP cells) [15]. These LEAP neurons express both DIMM and PHM, an enzyme required for alpha-amidation of neuropeptides [32], and have large cell bodies and/or extensive arborizations and axonal processes. Many peptide-expressing neurons were found not to co-express DIMM (non-LEAP cells) [15] and we indicate that the sNPF producing neurons in the larva are of this kind. Using the c929-GAL4 as a marker for *dimm *expression [24] and antiserum to the sNPF precursor we found no neurons displaying coexpression in the larval CNS. We hade expected a set of 3 pairs of LNCs displaying sNPF and corazonin immunoreactivity to co-express DIMM, but this was not the case. In the paper by Park et al. [15] a set of five pairs of neurons were shown to co-express DIMM and sNPF (antiserum to sNPF-2) in the larval brain. Four of these have cell bodies medially and colocalize dromyosuppressin (DMS) an FLRFamide-like peptide and another set of six are located among the LNCs and coexpress the neuropeptide corazonin. Since these authors used an antiserum to the full peptide sNPF-2 from Lee et al. [20] it is likely that the DMS expressing neurons cross react with the sNPF-2 antiserum (due to the shared RFamide). The corazonin-containing LNCs on the other hand may indeed colocalize sNPF and DIMM. It could be that we missed this colocalization in our preps while the c929-driven GFP was too weak in the cells of interest. As mentioned, we did reveal sNPFp and corazonin colocalization in three pairs of LNCs in the position described for the DIMM coexpressing ones [15] and if these neurons are true neurosecretory cells they would qualify as LEAP cells (however, see discussion below on relation to AKH cells).

The finding of sNPF exclusively (maybe with exception above) in small neurons that do not express DIMM suggest that the sNPF expressing neurons might be signaling locally and primarily in capacity of cotransmitter or local neuromodulator. Another neuropeptide gene, *dtk *and its DTK products, are also expressed mainly in small interneurons [12,56] and Park et al. [15] found that in the larva only one pair of *dtk *expressing neurons co-expressed DIMM. This pair of neurons also expresses allatostatin B (myoinhibitory peptide; MIP) and have extensive axonal projections all along the ventral nerve cord. Thus it is likely that both sNPF and DTKs are primarily cotransmitters and/or local neuromodulators.

### Colocalization of sNPF and other neurotransmitters

To seek support for a role of sNPFs in cotransmission we screened for colocalized neurotransmitters in the large population of sNPF immunoreactive neurons. In the mammalian nervous system neuropeptides colocalize extensively with classical neurotransmitters in interneurons [47,48,57], but in insects systematic screens of such colocalizations have not been performed (see [16]). Here, for simplicity, we used Gal4 expression for marking possible neurotransmitter phenotypes. This choice was partly forced by the fact the available antisera to small molecule neurotransmitters or their biosynthetic enzymes require fixation protocols incompatible with sNPF detection, or do not label neuronal cell bodies and thus precludes definite localization to the same neuron. Immunolabeling with sNPFp was made on Gal4-lines likely to reveal neurons expressing GABA, glutamate, acetylcholine, dopamine and octopamine/tyrosine. In this screen we did detect some patterns of colocalization between sNPF and GABA, glutamate and acetylcholine, but the majority on the sNPFp-IR interneurons did not colocalize the markers tested so far. The main findings on colocalized markers are shown in Table 1.

The antennal ORNs are likely to be cholinergic[58] and can thus be displayed by a *Cha*-Gal4 [33,34]. We found that all sNPF expressing ORNs in the antennae indeed coexpressed *Cha*-Gal4-driven GFP. The presence of an identified neuropeptide in arthropod chemosensory neurons is a novel finding, although antisera to FMRFamide-like peptides have been reported to label sensory axons in blowflies, locusts and lobsters [59-61]. Especially intriguing is the finding that only a subpopulation of the ORNs in the antenna and their axon terminations in the glomeruli in the antennal lobe express sNPF. Thus, most, if not all, ORNs are cholinergic, but only a subpopulation employ a putative peptidergic cotransmitter. It will be of interest to identify the complete set of glomeruli receiving sNPF-IR axons of ORNs to determine for which odors additional peptidergic signaling is utilized.

We detected a number of further *Cha*-Gal4 expressing neurons that colocalized sNPF in the larval and adult brain, but very few in the ventral nerve cord. Both in the brain and the ganglia these were primarily small interneurons that could not be individually identified, except for a pair of dorsal median neurons (DP) in A1 of the ventral nerve cord. When analyzing sNPF in relation to *Cha *expression in neuronal processes in brain neuropils we could not detect colocalization of markers in central body neuropils, mushroom bodies, optic lobe (with one exception in the lobula; Fig. 10H), whereas in the antennal lobes the ORN terminations coexpressed the two. Thus cholinergic interneurons that express sNPF appear to arborize primarily in non-glomerular neuropils.

Fewer sNPF expressing neurons displayed markers for glutamate (vGluT-Gal4) and GABA (Gad1-Gal4). These neurons were in both cases located in the brain and no colocalization was detected in the larval ventral ganglia. Again the neurons were not possible to identify as individuals and we did not see any specific patterns of colocalization in processes in major neuropils. We detected no colocalization between sNPF and markers for dopamine and octopamine/tyramine. However, we did not investigate the possible colocalization of sNPF and the biogenic amines serotonin and histamine, known to be expressed in distinct sets of neurons in the larval and adult CNS of *Drosophila *[62-65].

In summary, except for the ORNs of the antennae and abdominal DP neurons, it is not clear what specific types of interneurons that coexpress the sNPF and the markers for small molecule neurotransmitters. By screening labeling in different neuropil regions of the adult brain we could exclude peptide/transmitter coexpression in the neurons of the central body, antennal lobes (except ORNs), optic lobe and intrinsic neurons (Kenyon cells) of mushroom bodies. The Kenyon cells are likely to express a small molecule transmitter in addition to sNPF. A screen for such a transmitter was made in a previous paper [21] but no marker employed so far provided a lead to the Kenyon cell transmitter. Several putative small molecule transmitters remain to be investigated in *Drosophila *and other insects (e. g. nucleotides/nucleosides, glycine, and aspartate). It cannot be excluded that the small proportion of sNPF neurons where we could see colocalized markers was caused by deficiencies in the Gal4 lines utilized: the gene expressions may be incomplete and thus not reveal the entire populations of neurons expressing the neurotransmitters intended.

### Relation between sNPF and other neuropeptides, including ILPs

Since we were mainly interested in the colocalization of sNPF and small molecule transmitters we did not make an extensive screen for colocalization with other neuropeptides.

One exciting finding was that the two unique DP neurons of A1 of larvae co-express the relaxin-like ILP7, as well as *Cha*. These neurons were described first as ILP7 producing neurons with ascending axons terminating close to the dendrites of the ILP producing MNCs in the larval brain [41]. Here we showed that the ascending ILP7 immunoreactive processes indeed colocalize sNPF and are part of a slightly more extensive supply of sNPF-IR processes impinging on Dilp2-Gal4 expressing MNCs. It was shown that sNPF overexpression causes increased production of ILPs in these MNCs and that the MNCs express the sNPF receptor [31]. Thus, it is possible that sNPF together with ILP7 (and maybe acetylcholine) regulate production of other ILPs. It should be noted that we also could show that sNPF immunolabeled varicose axons (from the three pairs of LNCs) terminate close to AKH-producing cells in the corpora cardiaca of the ring gland. This might suggest that these sNPF producing cells could have a role in regulation of the AKH cells, but it cannot be excluded that sNPF is released as a circulating hormone. The same LNCs have additional axons terminating in the anterior aorta suggesting a hormonal release site.

Apart from the above findings we revealed one pair of neurons in the dorsal protocerebrum (in DLN1 group) co-expressing sNPF and NPF (long NPF) and noted an extensive superposition of neuronal processes containing he two peptides in the dorsal protocerebrum. Another set of about six sNPF-IR neurons in the dorso-median brain (pars intercerebralis) co-express Mai-179-Gal4. These particular neurons with axons in the median bundle and possible terminations in tritocerebrum were not described among the neurosecretory cells innervating the ring gland in *Drosophila *[27], but similar neurons were seen with an antiserum to Ion transport peptide (ITP) [66]. Thus, it might be that these PI neurons coexpress ITP and sNPF.

### How many peptides are produced by the *snpf *gene?

Mass spectrometry identified SPSLRLRFa, which corresponds to both sNPF-1^4–11 ^and sNPF-2^12–19^, as the major product of the predicted sNPF-1 to 4. A small peak representing full length sNPF-1 was also detected. Thus, it appears that sNPF-2 is processed to sNPF-2^12–19^, and sNPF-3 and 4 are not liberated from the precursor (see also [43-45]). An antiserum to sNPF-3 used in a previous study [21] labeled the same neurons as the one to the sNPF precursor used here. Possibly that antiserum recognizes the sNPF-3 and 4 sequences (with RLRWamide) within the precursor, or too little peptide is produced to be detected by mass spectrometry. The *snpf *genes of the mosquitoes *Anopheles gambiae, Aedes aegypti*, and *Culex pipiens *also predict RLRWamides like sNPF-3 and 4, but not the ones in the honey bee or the red flour beetle *Tribolium *[50,53,54]. We propose that the neurons we depicted with the sNPFp antiserum and ribonucleotide probe express at least one peptide derived from the *snpf*.

### Functions of sNPF: diverse or not?

Judging from the abundance of sNPF distribution in diverse sets of neurons in the larval and adult CNS one would anticipate multiple functions of these peptides. An earlier study has addressed the general distribution and role of sNPFs and its receptor sNPFR1 in feeding and growth in *Drosophila *[20,31]. These authors showed that sNPF signaling appears to be involved in regulation of food intake and growth, [20], and also in the regulation of *Drosophila *ILP expression via the sNPF receptor [31]. The experiments were conducted by rather widespread over-expression and knock-down of sNPF products and did not address sNPF action in specific circuits. Thus, it is not clear at what level the sNPF regulation of ILP producing cells occurs. However, the sNPFR1 is expressed on Dilp2-gal4 expressing MNCs in brain and sNPF immunoreactive processes were detected close to these cells [31], as also shown here. Whatever the exact circuitry utilizing sNPF to regulate ILP production, the effect of sNPF on growth via ERK (extracellular signal-related kinases) signaling is likely to be only a part of a spectrum of sNPF functions in *Drosophila*. This function may be limited to a small subset of the sNPF-expressing neurons.

Peptides likely to be orthologs of *Drosophila *sNPF have been identified in other insects. In fact the first identification of an sNPF (or *Aedes *head peptides) was in the mosquito *Aedes aegypti *[67,68] and subsequently related peptides have been identified in several other insect species [50,53,69,54,72]. Some actions of sNPF have been analyzed by peptide administration: sNPFs are myostimulatory on various visceral muscles [68,71] induce host seeking behavior in female mosquitoes [73], they may be important for diapause in a beetle [74] and in locusts they stimulate ovarian growth and induce increases in vitellogenin levels in the circulation [71,75]. This spectrum of actions, except the host seeking behavior, may represent hormonal functions of sNPF. Thus, probably a host of functions based on local actions of sNPF in circuits of the CNS remain to be demonstrated.

Only a single *Drosophila *sNPF receptor (NPFR76F; CG7395; sNPFR1) has been identified [76-78]. This receptor may couple to different signaling pathways, as already suggested from in vitro experiments [31,76-78]. It has been postulated that the sNPFR1 is related to the mammalian neuropeptide Y type of receptors [76,77] and the receptor for *Drosophila *long NPF [51,79]. Long NPF is encoded on a separate gene (*npf*), identified in *Drosophila *and other insects [51,52,80]. This peptide is 36 amino acids in length with an ARVRFamide C-terminus and resembles neuropeptide Y of mammals. In spite of the unfortunate similarities in names of the two peptide genes and peptides, they are part of two functionally distinct signaling systems [20,21,31,39].

## Conclusion

The diverse distribution of sNPF in *Drosophila *suggests the following functions: A role in olfaction at the level of the first synapse between ORNs and second order neurons in the antennal lobe, a role in olfactory processing (or olfactory learning, or any of the other MB roles) at the level of the mushroom bodies, neuromodulation in central body circuits possibly involved in locomotor control, roles in regulation of hormonal systems (ILPs and AKH producing cells) or even as circulating neurohormones and finally sNPF seem to have modulatory/cotransmitter functions in numerous circuits in the brain and ventral nerve cord. Colocalized putative neurotransmitters as well as neuropeptides have been indicated in several subsets of sNPF expressing neurons and a more complete screen is likely to detect further examples. The colocalized substances may be both excitatory (e. g. acetylcholine) and inhibitory (GABA) or neuropeptides with various modulatory or hormonal actions. Clearly more specific interference with sNPF signaling in different neuron systems is required to begin to understand the functional diversity of this peptide.

## Methods

### Fly strains used

We used wild type (Oregon R) and white eyed (*w*^1118^) fruitflies, *Drosophila melanogaste*r for immunocytochemistry and *in situ *hybridization. A number of Gal4 strains were used for driving GFP to identify specific sets of neurons in relation to sNPF markers. These were: *gad1*-Gal4 (Glutamic acid decarboxylase-1 promoter-Gal4 [81]; from G. Miesenböck, New Haven, CT), *Cha*-Gal4 (w*; P{Cha-GAL4.7.4}19B P{UAS-GFP.S65T}T2; choline acetyltransferase promotor-Gal4-GFP fusion, from Bloomington *Drosophila *Stock Center at Indiana University, IN [33]); *th*-Gal4 (tyrosine-hydroxylase promoter-Gal4; [38]; from S. Birmann, Marseille, France), *tdc*-Gal4 (tyrosine decarboxylase promoter Gal4; obtained from J. Dubnau, Cold Spring Harbor, NY; [82]), OK371-Gal4 (vesicular glutamate transporter-Gal4) (from H. Aberle, Tübingen, Germany [36]; OK107-Gal4 (P{GawB}OK107 (expression in mushroom body neurons); from Bloomington Stock center; [83,84]), *npf*-Gal4 (Neuropeptide F-promoter-Gal4; from P. Shen, Athens, GA; [39]). A snpf-Gal4 (NP6301; order number 113901) was obtained from the *Drosophila *Genetic Resource Center (DGRC), Kyoto Institute of Technology, Kyoto, Japan. The genotype of this Gal4 is yw; P{GawB}NP6301/CyO, P{UAS-lacZ.UW14}UW14. To our knowledge the expression of this Gal4 has not been previously described. The enhancer trap line Mai179-Gal4 [27] was obtained from G. Korge (Berlin, Germany). This drives expression in a subset of neurons in the brain including some neurosecretory cells. The c929-Gal4 line was obtained from P. H. Taghert (St. Louis, MO). This Gal4 reveals the *Drosophila dimmed *(*dimm*) gene that encodes a bHLH protein, DIMM, known to be required for the differentiation of peptidergic neurons and endocrine cells [24]. Finally, UAS-cd8gfp flies (from Bloomington Stock center) were used as targets to express GFP.

### In situ hybridization

The coding region of the *snpf *gene (0.85 kb) subcloned into a pGEM-T easy vector (Promega, Madison, WI) was kindly provided by Dr. K. Yu (Korea Research Institute of Bioscience and Biotechnology, Daejeon, Korea;[20]). This was amplified with polymerase chain reaction (PCR) and the PCR product was cut with either APA1 (for antisense strand) or *Pst*1 (for sense strand) and purified. An antisense digoxigenin (Dig)-labeled *snp *probe was generated with SP6 RNA polymerase and Dignucleotide (NTP/Dig-UTP) mixture (Roche Diagnostics, Indianapolis, IN). As a control we generated an *snpf *sense probe with T7 polymerase. In situ hybridization on whole mount larval CNS and adult brains (at least 10 specimens of each stage) with the Dig-labeled *snpf *riboprobes (100 ng/l) was performed according to Johard et al. [21]. In brief, tissues were fixed in 4% paraformaldehyde (PFA) in 0.1 M sodium phosphate buffer (PB) and 0.1% Tween 20 (PBT) for 30 minutes at room temperature, washed in PBT and incubated for 10 minutes in 20 g/ml proteinase K (Sigma, St. Louis, MO) in PBT, at 37°C. A postfixation was made for 20 minutes in 4% PFA. Prehybridization was performed for 1 hour at 55°C in hybridization buffer consisting of 50% formamide, 5× SSC, 140 mM NaCl, 10 mM KPO4, 0.1 mg/ml glycogen, 100 g/ml heat-denatured herring sperm DNA (Sigma), and 0.1% Tween 20. Hybridization was carried out for 48 hours at 55°C. After thorough washes anti-Dig immunocytochemistry was performed on the tissues, starting by blocking in 4% normal goat serum (NGS) or 1% milk powder in PBT. Alkaline phosphatase (AP) tagged anti-Dig-antiserum (1:1,000) was added for 2 hours, and after washes AP histochemistry was performed in nitroblue tetrazolium/5-bromo-4-chloro-3-indolyl phosphate (NBT/BCIP) solution (Roche Diagnostics, Mannheim, Germany) for about 1 hour. After washes, tissues were mounted under coverslips in glycerol/PB (80%).

### Antisera used

We initially used antisera raised to three peptides predicted from the *snpf *precursor [21]: (1) SPSLRLRFamide, expected to recognize the sNPF-1 and sNPF-2, (2) PQRLRWamide, expected to recognize sNPF-3 and sNPF-4, and (3) DPSLPQMRRTAYDDLLEREL, which is part of the *snpf *precursor (sNPFp) and not likely to be a bioactive peptide [21]. These antisera were kindly provided by Dr. J. A. Veenstra (Bordeaux, France). The peptides were coupled with 1,5 difluoro-2,4-dinitrobenzene to bovine serum albumin for immunization in rabbits, and antisera were raised in two New Zealand white rabbits for each antigen. The three peptide sequences used for immunization were also employed for preabsorption of antisera in immunocytochemical specificity tests. The working dilution for primary antisera in the experiments were as follows: anti-SPSLRLRFamide at 1:1000, anti-PQRLRWamide at 1:1000 and anti-DPSLPQMRRTAYDDLLEREL at 1:4000. Throughout the present paper we only used the sNPFp antiserum for imaging.

A rabbit antiserum to the neuropeptide corazonin (kind gift from Dr. J. A. Veenstra, Bordeaux, France) was used at 1:2000 (see [28]). A rabbit antiserum to the B-chain of *Drosophila *insulin-like peptide 7 (ILP-7), used at 1:5000, was a kind gift from Dr. I. Miguel-Aliaga (London, UK) [41]. Mouse monoclonal antibodies to fasciclin-2 (anti-Fas2; mAb 1D4) were obtained from the Developmental Studies Hybridoma Bank (University of Iowa, IA) and used at 1:75). These were used for double labeling with sNPF antisera to provide a correlation to known axonal tracts (see [7,25]). A mouse monoclonal antibody to GFP (mAb 3E6; code #A-11120; Molecular Probes, Leiden, Netherlands) was used at 1:1000 for amplifying the GFP signal in some specimens.

### Immunocytochemistry

For most purposes opened head regions of feeding third-instar larvae of opened adult heads were fixed for two hours in 4% paraformaldehyde (PFA) in 0.1 M sodium phosphate buffer (PB; pH 7.4) on ice. Following rinsing in PB, the central nervous system was dissected out and preincubated over night in incubation buffer consisting of 0.01 M phosphate-buffered saline, pH 7.4, with 0.25% BSA and 0.25% Triton-X and 3% Normal Goat Serum. Incubation with primary antisera were performed for 48 h in dark at 4°C with gentle agitation followed by thorough rinses in PBS with Triton-X at room temperature. The primary antibodies were detected with Cy3-conjugated goat anti-rabbit antiserum (Jackson ImmunoResearch, West Grove, PA) or Cy2- or Cy3-conjugated goat anti-mouse antiserum (Jackson ImmunoResearch) all used at 1:1500 or 1:1000. Some specimens were processed for peroxidase based detection using the biotin-streptavidine method (with peroxidase histochemistry) employing a commercial ABC kit (Vectastain) from Vector Laboratories (Burlingame, CA) according to the instructions supplied by the manufacturers.

For cryostat sections fixed heads were put in 20% sucrose over night and subsequently frozen at -23°C and cut at 20 μm on a cryostat (Leitz, Wetzlar, Germany). Sectioned tissues were subjected to the same immunocytochemical procedure as above. Preincubations of antisera with their respective antigens and other specificity tests for immunocytochemistry were described previously [21].

### Image analysis

Specimens were imaged with a Zeiss LSM 510 (Zeiss, Jena, Germany) or a Leica TCS SP2 (Leica Microsystems, Wetzlar, Germany) confocal laser scanning microscope. Confocal images were obtained at an optical section thickness of 0.4 – 0.5 μm and were processed with Zeiss LSM or Leica Confocal Software and edited for contrast and brightness in Adobe Photoshop CS3 Extended.

To obtain 3D reconstructions, confocal stacks were subjected to either texture-based volume rendering using the voltex tool, or to threshold-based surface generation with the LabelVoxel and Threshold Segmentation tool of AMIRA 4.1 software (Mercury Computer Systems GmbH, Berlin, Germany).

Wholemounted specimens treated for in situ hybridization or peroxidase-based immunocytochemistry were used for tracings of neuronal cell bodies with a drawing tube attached to a compound microscope. Images of in situ hybdridization were generated on a Zeiss Axioplan 2 microscope equipped with a Zeiss Axiocam HRM CCD camera.

### Mass spectrometry

The brain of either larvae or adults was dissected free from all surrounding tissue in standard *Drosophila *saline. Optic anlagen or lobes and the ring gland/aorta were removed with fine scissors. The remaining brain was cut transversally in the middle. The dorsal part, which roughly corresponded to the protocerebrum, was then slightly trimmed dorsally and laterally, and cut medially. The resulting tissue fragments each contained a mushroom body, as checked under a phase contrast microscope. The fragments were transferred to a stainless steel MALDI target and attached saline was aspirated off, and the preparation was left to dry. Afterwards, nanoliter volumes of matrix (saturated solution of recrystallized α-cyano-4-hydroxycinnamic acid (CHCA, Sigma-Aldrich) in 30% MeOH/30% EtOH) were added to the samples using a manual oocyte injector (Drummond Digital).

MALDI-TOF mass spectra were acquired in positive ion mode on a Applied Biosystems 4800 *plus *mass spectrometer (Applied Biosystems, Darmstadt, Germany). Samples were analyzed in reflectron mode after calibration with synthetic peptides. To suppress matrix signals, the low mass gate was set to 800 Da. Laser power was adjusted to provide optimal signal-to-noise ratios. Data were analyzed using Data Explorer 4.3 software (Applied Biosystems), with a mass tolerance of 0.1 Da.

## Authors' contributions

DRN and HADJ designed the experiments, LE and HADJ carried out the bulk of the antibody, Gal4-GFP and in situ labeling, JGS and CW performed some of the immunolabeling and all Amira reconstructions, CW performed mass spectrometry analysis and produced the movies, DRN provided reagents, and all authors analyzed various portions of the data, and read, discussed and approved the paper.

## Supplementary Material

Additional file 1 Rotating view of sNPFp-IR neurons in the ventral nerve cord of a third instar larva. Click here for file

Additional file 2 Rotating view of sNPFp-IR neurons in the brain of a third instar larva. The most prominent labeling is seen in the mushroom body lobes. For further details see Fig. 2D. Click here for file

Additional file 3 Direct MALDI-TOF profiling of protocerebrum fragments containing the mushroom bodies.**A – C **Direct peptide profiling in larva (**A**) and adult (**B – C**) brain tissue. Typically, the most intense mass peak corresponded to the theoretical mass of sNPF-1^4–11 ^or sequence-identical sNPF-2^12–19 ^with the sequence SPSLRLRFa (labeled sNPF, theoretical monoisotopic mass 974.60 Da). In one out of ten preparations, a small mass peak corresponding to the full length sNPF-1 was found (**B**). Other predicted sNPF-peptides could not be detected. **D **MALDI-TOF-TOF fragmentation of the mass peak at 974.6 from adult tissue. The measured b and y fragments clearly indicate that this mass peak corresponds to SPSLRLRFa, although the parent material was too small to obtain a full fragment ion series. Click here for file
