# Supplementary figures and images for: A large population of diverse neurons in the Drosophila central nervous system expresses short neuropeptide F, suggesting multiple distributed peptide functions
Source: BMC Neurosci. 2008 Sep 19;9:90. doi: 10.1186/1471-2202-9-90 (PMC2569041; doi:10.1186/1471-2202-9-90)

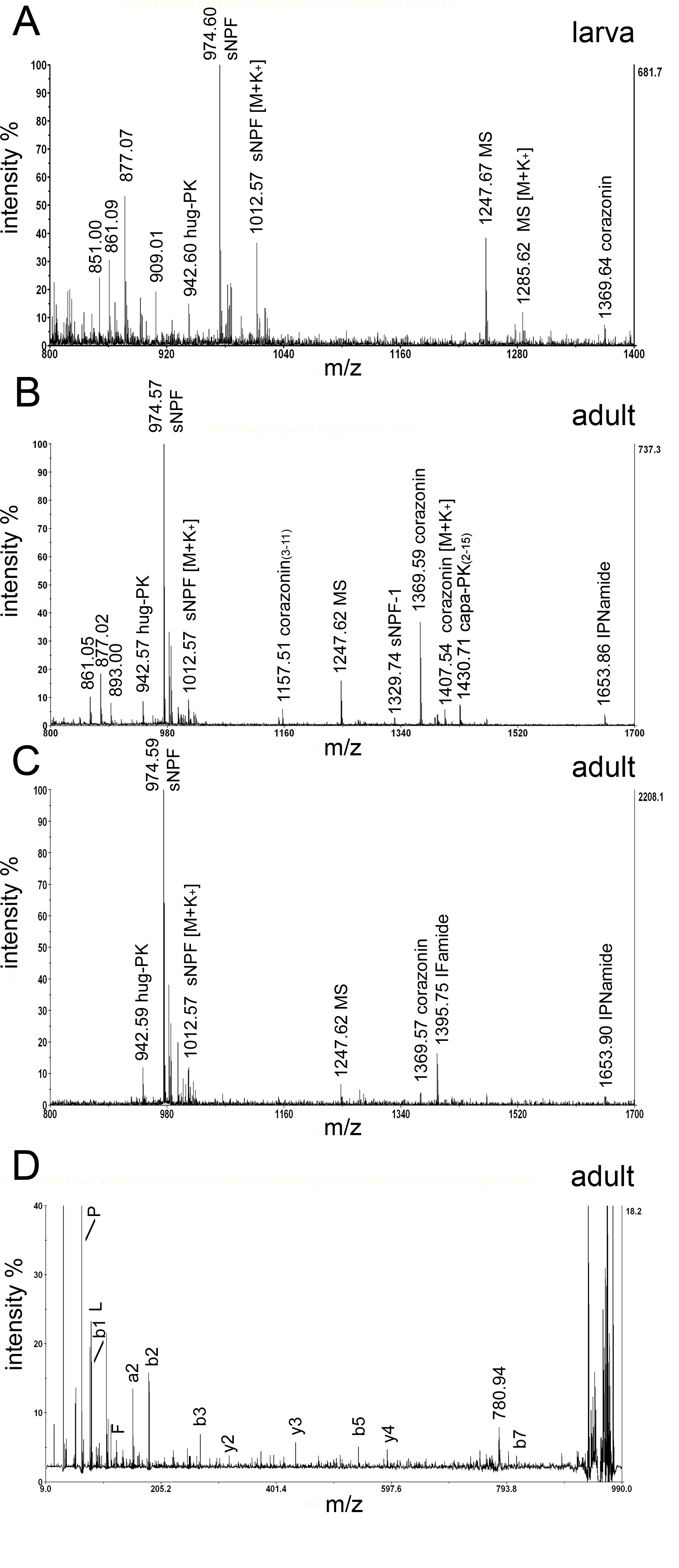

Supplement: Additional file 3 — Direct MALDI-TOF profiling of protocerebrum fragments containing the mushroom bodies.A – C Direct peptide profiling in larva (A) and adult (B – C) brain tissue. Typically, the most intense mass peak corresponded to the theoretical mass of sNPF-14–11 or sequence-identical sNPF-212–19 with the sequence SPSLRLRFa (labeled sNPF, theoretical monoisotopic mass 974.60 Da). In one out of ten preparations, a small mass peak corresponding to the full length sNPF-1 was found (B). Other predicted sNPF-peptides could not be detected. D MALDI-TOF-TOF fragmentation of the mass peak at 974.6 from adult tissue. The measured b and y fragments clearly indicate that this mass peak corresponds to SPSLRLRFa, although the parent material was too small to obtain a full fragment ion series. [file 1471-2202-9-90-S3.tiff]
